# Binding dynamics of a monomeric SSB protein to DNA: a single-molecule multi-process approach

**DOI:** 10.1093/nar/gkv1225

**Published:** 2015-11-17

**Authors:** Michael J. Morten, Jose R. Peregrina, Maria Figueira-Gonzalez, Katrin Ackermann, Bela E. Bode, Malcolm F. White, J. Carlos Penedo

**Affiliations:** 1 Biomedical Sciences Research Complex, University of St Andrews, St Andrews, Fife KY16 9ST, UK; 2 EaStCHEM School of Chemistry and Centre of Magnetic Resonance, University of St Andrews, St Andrews, Fife KY16 9ST, UK; 3 SUPA School of Physics and Astronomy, University of St Andrews, St Andrews, Fife KY16 9SS, UK

## Abstract

Single-stranded DNA binding proteins (SSBs) are ubiquitous across all organisms and are characterized by the presence of an OB (oligonucleotide/oligosaccharide/oligopeptide) binding motif to recognize single-stranded DNA (ssDNA). Despite their critical role in genome maintenance, our knowledge about SSB function is limited to proteins containing multiple OB-domains and little is known about single OB-folds interacting with ssDNA. *Sulfolobus solfataricus* SSB (SsoSSB) contains a single OB-fold and being the simplest representative of the SSB-family may serve as a model to understand fundamental aspects of SSB:DNA interactions. Here, we introduce a novel approach based on the competition between Förster resonance energy transfer (FRET), protein-induced fluorescence enhancement (PIFE) and quenching to dissect SsoSSB binding dynamics at single-monomer resolution. We demonstrate that SsoSSB follows a monomer-by-monomer binding mechanism that involves a positive-cooperativity component between adjacent monomers. We found that SsoSSB dynamic behaviour is closer to that of Replication Protein A than to *Escherichia coli* SSB; a feature that might be inherited from the structural analogies of their DNA-binding domains. We hypothesize that SsoSSB has developed a balance between high-density binding and a highly dynamic interaction with ssDNA to ensure efficient protection of the genome but still allow access to ssDNA during vital cellular processes.

## INTRODUCTION

In all cellular organisms, the genome is organized as a double stranded DNA helix (dsDNA) with the nucleotide bases carrying the genetic information sequestered in the interior of the double strand, protected from damaging agents (1). To provide access to genomic information during vital cellular processes such as DNA replication, transcription, recombination and repair, dsDNA must be unwound to produce single stranded DNA (ssDNA) (2). Although transient exposure of the genetic information is crucial for cell survival, the presence of ssDNA intermediates increases the possibility of chemical and physical damage, thus compromising genome stability (2,3). To preserve ssDNA integrity and still facilitate DNA processing, single-stranded DNA binding proteins (SSBs, also known as Replication Protein A, RPA, in eukarya) interact with DNA in a sequence-independent manner (4). In addition to protecting ssDNA from degradation, the high affinity of SSBs has also been exploited by nature to detect DNA damage (2, 5–8), melt dsDNA (5–7) and to recruit other proteins to ssDNA stimulating their biochemical activity and the overall efficiency of DNA processing pathways (9–12). The vital roles of SSBs in genome maintenance and cell survival are reflected in their universal expression throughout all kingdoms of life (3, 13–15), as well as their inclusion in some viral proteomes (15). In recent years, there has been also an increasing interest in the biotechnological potential of SSBs (16). A number of studies have reported the use of SSBs, mostly of thermophilic origin due to their higher stability, to improve the efficiency and specificity of the polymerase chain reaction (PCR) (17, 18).

Although very little conservation has been found at sequence level, SSB proteins across all domains of life exhibit some evolutionary traits since their high affinity for ssDNA comes largely from the presence of a distinctive and commonly shared ‘OB-fold’ motif (oligonucleotide/oligosaccharide binding fold) (19, 20). As observed in the crystal structures of human (20) and fungal RPA (21), *Escherichia coli* SSB (*EcoSSB*) (22, 23), and archaeal SSB (24), these OB-fold domains comprise ∼100 amino acids and are typically organized as a five β sheet barrel (25, 26). The presence of a structurally conserved SSB binding fold across all domains of life suggest that SSBs are derived from a common evolutionary ancestor protein through gene duplication, merging and reshuffling events (25). In addition to this ubiquitous N-terminal OB motif, many SSBs have a C-terminal flexible tail that is thought to function as recognition sequences for protein–protein interactions (25–27).

The subunit composition and oligomeric state of SSB proteins, show remarkable variability over the three domains of life (4). The oligomeric state of bacterial SSB proteins is exemplified by the homotetrameric organization of the *Eco*SSB, which has served as the prototypical SSB protein for many years (22, 23). In *Eco*SSB each monomer encodes for a single OB-fold, but exceptions to this arrangement include SSBs composed of two OB-folds per monomer and acting as homodimers in *Deinococcus-thermus* genera (28). Outside of the bacteria, SSBs exhibit quaternary structures ranging from the heterotrimeric organization of the eukaryotic RPA (29) to the dimeric and monomeric forms of several bacteriophage and viral SSB (15, 30). An heterotrimer OB scaffold with four DNA-binding OB domains is present also in euryarchaea such *Pyrococcus furiosus* (31). In contrast, the SSB from the crenarchaeote *Sulfolobus solfataricus* (SsoSSB) shows a single RPA-like OB domain and a C-terminal tail resembling that of *EcoSSB* (24, 32). It has been demonstrated that SsoSSB can specifically melt double helices carrying a range of lesions including mismatches, adducts and photoproducts (6). Such potential to discriminate *in vitro* between damaged and non-damaged DNA suggests a potential role for SsoSSB in sensing DNA damage. The C-terminal tail of SsoSSB has been shown to mediate interactions with RNA polymerase (11), reverse gyrase (33) and the NurA nuclease (12).

In recent years, the study of the interaction between SSB proteins and ssDNA using single-molecule techniques has emerged as an unique method to provide information not accessible by any other approach (26, 34, 35). Most of these studies have focused on characterizing the transition dynamics between the different binding modes of *EcoSSB* (34–36) to DNA using single-molecule fluorescence resonance energy transfer assays (sm-FRET) (37). Although these studies have provided remarkable insights into how the binding modes of bacterial SSBs dynamically interconvert depending on nucleotide length and environmental conditions, they have focused on SSB proteins containing multiple OB-fold domains. Therefore, the interaction mechanism between monomeric SSBs and ssDNA remains unclear and this raises the question of how much of our current knowledge from multimeric SSBs is preserved down to the single OB-fold level. This is important because in addition to RPA as the major SSB protein in eukaryotes, SSBs containing a single OB-fold have recently been discovered and shown to play a critical role in genomic stability and the repair of double strand breaks (9, 38).

Here, we have taken advantage of the single-OB organization of SsoSSB and use a novel single-molecule fluorescence-based multi-process approach to delineate, for the first time, the dynamics of individual OB-folds interacting with ssDDNA. Using Alexa647-labelled SsoSSB (A647-SsoSSB) and Cy3-labelled ssDNA, we show that the close positioning of the labelled monomers on the ssDNA leads to a range of photophysical events including Förster resonance energy transfer (FRET), protein-induced fluorescence enhancement (PIFE) and acceptor quenching within the same single-molecule trajectory that can be exploited to dissect SSB dynamics. FRET has been extensively used as a molecular ruler to measure conformational changes within biomolecules and biomolecular interactions (37), whereas the increase in the quantum yield of fluorescent molecules due to the presence of proteins in the close proximity (PIFE) has been recently exploited as a method to investigate binding dynamics. Through a combination of ensemble-averaging fluorescence techniques and single-molecule measurements, we further demonstrate that each of these processes can be unambiguously assigned to a specific binding step during the DNA-coating process, thus allowing us to visualize in real-time the binding mechanism at single monomer resolution. Using this approach, we determine that monomeric SsoSSB is the functional species interacting with single-strand DNA in solution and observe evidence for a significant degree of cooperative interaction between adjacent SSB monomers along the DNA strand. Taken together, our results strongly indicate that SsoSSB binding to ssDNA is a highly dynamic process taking place following a monomer-by-monomer association mechanism involving a key cooperative step between adjacent monomers.

## MATERIALS AND METHODS

### Protein expression and purification

Wild-type SSB and variants A114C from *S. sulfolobus* were transformed by addition of glycerol stocks of DNA to 50 μl of C43 competent cells, plated on to agar plates containing kanamycin and incubated overnight at 37°C. A single colony was extracted and grown overnight in LB broth with kanamycin (35 μg/ml). IPTG was introduced to a final concentration of 0.4 mM to induce expression of SSB for a further 4 h being agitated at 37°C. The cells were pelleted by centrifugation (Beckmann, JLA 8.1000 rotor) at 4°C, 5000 rpm for 20 min and frozen at −20°C until required. Lysis buffer was used to re-suspend the thawed pellet up to a volume of 50 ml and immediately sonicated (Soniprep 150, MSE (UK) Ltd) at 4°C. The pellet was heat treated at 70°C for 20 min and the denatured proteins were precipitated by centrifugation (Beckman, JA 25.50 rotor) at 4°C, 20 000 rpm for 20 min. The supernatant was diluted with an equivalent volume of buffer A (20 mM Tris–HCl (pH 7.40), 1 mM ethylenediaminetetraacetic acid (EDTA) and 1 mM Dithiothreitol (DTT)) to reduce the NaCl concentration below 250 mM and filtered (Millex, 0.22 μm). The solution was passed through two 5 ml heparin-sepharose column (GE healthcare) equilibrated with buffer A and eluted off the column along a concentration gradient of buffer B (20 mM Tris–HCl (pH 7.40), 1 mM EDTA and 1 mM DTT and 1M NaCl) and the protein was collected in fractions. The fractions containing protein were concentrated down to below 10 ml and passed through a HiLoad 26/60 Superdex 200 size exclusion column (GE Healthcare) equilibrated with gel filtration buffer. To remove any remaining impurities, the protein was passed through a 5 ml sepharose column (GE Healthcare) equilibrated with buffer A, and eluted off with buffer B. The protein concentration in each fraction was monitored using UV-vis adsorption at 260 nm and an extinction coefficient of 12660 M^−1^ cm^−1^ and proteins peaks were investigated using a SDS-PAGE gel (Invitrogen, 200 V for 35 min).

### SsoSSB labelling and purification

To allow site specific labelling of SsoSSB with fluorescent or spin labels, a cysteine was introduced at residue 114 using site-directed mutagenesis. This was performed using standard protocols (QuikChange, Stratagene). The purified A114C SSB mutant was labelled with Alexa Fluor 647 C2 maleimide (Life Technologies) according to the manufacturer's instructions. Unlabelled and labelled proteins were separated by eluting the mixture through a SP-Sepharose High Performance 26/10 affinity column as described in the Supplementary Methods section. The labelling efficiency was checked by MALDI-TOF mass spectrometry and UV-vis spectrometry and a value of 99% was obtained. To increase the yield of the labelling reaction, the SSB was unfolded by adding 8 M urea to the labelling mixture thus exposing the reactive thiol. This was diluted down to 2 M urea with buffer A so the protein could re-fold before passing through the affinity column. Purified A114C SsoSSB was also labelled with 1-Oxyl-2,2,5,5-tetramethylpyrroline-3-methyl) a thiol-specific methanethiosulfonate spin label MTSSL (Toronto Research Chemicals) to yield the spin-labelled A114R1 SsoSSB as described in the literature (39).

### DNA labelling and purification

Oligonucleotides were purchased from Integrated DNA Technologies, with 5′ biotin and 3′ amino group modifications. Succinimidyl ester derivatives of the fluorophores Cy3, Alexa647 (GE Healthcare) and Alexa 488 (Invitrogen) were used according to the manufacturer's protocol. Initially, the dry pellet of DNA was dissolved in deionized water to a concentration of 25 μg/μl. A total of 4 μl of this solution were added to 7 μl of deionized water and 75 μl of labelling buffer (0.1 M sodium tetraborate, pH 8.5) and incubated at room temperature overnight. The DNA was precipitated by adding 10 μl of 3M of sodium acetate and absolute ethanol was added, gently mixed and incubated overnight at −20. The mixture was then spun at 13 000 rpm for 1 h. The supernatant was removed and the pellet dissolved in 50 mM Tris–HCl pH 7.5. The DNA was separated from any unbound dye using a denaturing polyacrylamide gel 12% with 7 M urea, 300 μl of APS (ammonium persulfate) and 30 μl of TEMED. The gel was run for 1 h in Tris borate/EDTA buffer (TBE) before the DNA was loaded in 50% of formamide at 18 W with a set temperature threshold of 25°C. Following electrophoresis the band was cut from the gel, transferred to a vial and chopped and re-suspended in 400 ml 50 mM Tris pH 7.5. The DNA was incubated overnight wrapped in aluminium foil in a shaker. The DNA was then spun at 13 000 rpm for 10 min and the supernatant retrieved using a micro spin column. The concentration was measured on Cary Varian UV-vis spectrophotometer.

### Ensemble fluorescence

Ensemble experiments were performed in 50 mM Tris–HCl pH 7.6, 10 mM KCl, 5% glycerol, 0.1 mg/ml bovine serum albumin with 5–50 nM DNA substrate. Fluorescent titrations were performed on a Varian Cary Eclipse Fluorescence Spectrophotometer (Varian Inc., Palo Alto, USA) exciting the Cy3 at 550 nm, the Alexa647 at 640 nm and the tryptophan residues at 295 nm. PIFE titrations were carried out at 20°C and 65°C to determine the influence of temperature. FRET and acceptor quenching titrations were performed at 20°C. UV-vis spectra were recorded on a Varian Cary 50 Bio UV-visible Spectrophotometer (Varian Inc., Palo Alto, USA). PIFE, FRET and acceptor quenching isotherms where analysed as described in Supplementary Methods section.

### Single-molecule measurements

Single-molecule FRET and PIFE measurements were performed using a prism-type total-internal reflection (TIR) microscope (Olympus IX71). The sample was excited by a 50 mW CW laser operating at 532 nm (Crystalaser). The detector was a back-illuminated Ixon EMCCD camera (Andor, Belfast). Data were acquired in imaging buffer (50 mM Tris–HCl (pH 8.0), 50 mM NaCl, 6% (w/w) glucose, 1% 2-mercaptoethanol, 0.1 mg/ml glucose oxidase (Sigma) and 0.02 mg/ml glucose catalase (Sigma), 1 mM Trolox. Measurements were carried out at room temperature (20°C) with frame integration times of 50 ms unless stated otherwise. Images were processed using IDL and data analysis was performed using laboratory-written routines in Matlab as previously described (41). Apparent FRET efficiency after background correction, *E_app_* was calculated by (I_A_/[I_A_+I_D_]), where I_A_ and I_D_ are the fluorescence intensities of donor and acceptor, respectively. Single-molecule histograms were calculated using the first 15 frames of each trajectory. For the analysis of the photobleaching rate of Alexa Fluor 647, A647-SsoSSB was encapsulated in 100 nm diameter small unilamellar vesicles (SUV) prepared using the extrusion method as described previously (40). Vesicles were prepared by mixing eggPC and 16:0 biotinyl cap PE in 1:100 molar ratio in chloroform followed by 2 h evaporation in a dessicator and rehydration in a buffer (10 mM Tris–HCl, pH 7.5, 150 mM NaCl and 1 mM EDTA) containing 400 nM of A647-SsoSSB. The mixture was then extruded through 100 nm diameter pores (Avanti Mini Extruder) to form SUVs, store at 4°C and used within 1 week.

## RESULTS

### SsoSSB binding to ssDNA monitored by protein-induced fluorescent enhancement (PIFE)

It is well known that the emission intensity of cyanine and related dyes (i.e. Cy3, Cy5, DY547), positioned on a nucleic acid sequence, may become significantly increased when proteins bind in their close proximity (42). This effect, termed PIFE, has been attributed to local variations in the viscosity of the medium surrounding the dye that alter their cis-trans isomerization mechanism. In recent years, the application of PIFE at single-molecule level (smPIFE) has emerged as a complementary technique to Förster resonance energy transfer methods (smFRET). In addition to the advantage of bypassing protein labelling, PIFE provides a means to monitor the time-dependent variation in intermolecular distance between a fluorescently labelled oligonucleotide and a protein in the short distance range (0–3 nm), where FRET methods are rather insensitive. PIFE has already been employed to investigate filament-forming proteins such as RecA (42) and enzymes such as BamHI (42) and Fen1 (43).

**Figure 1.**
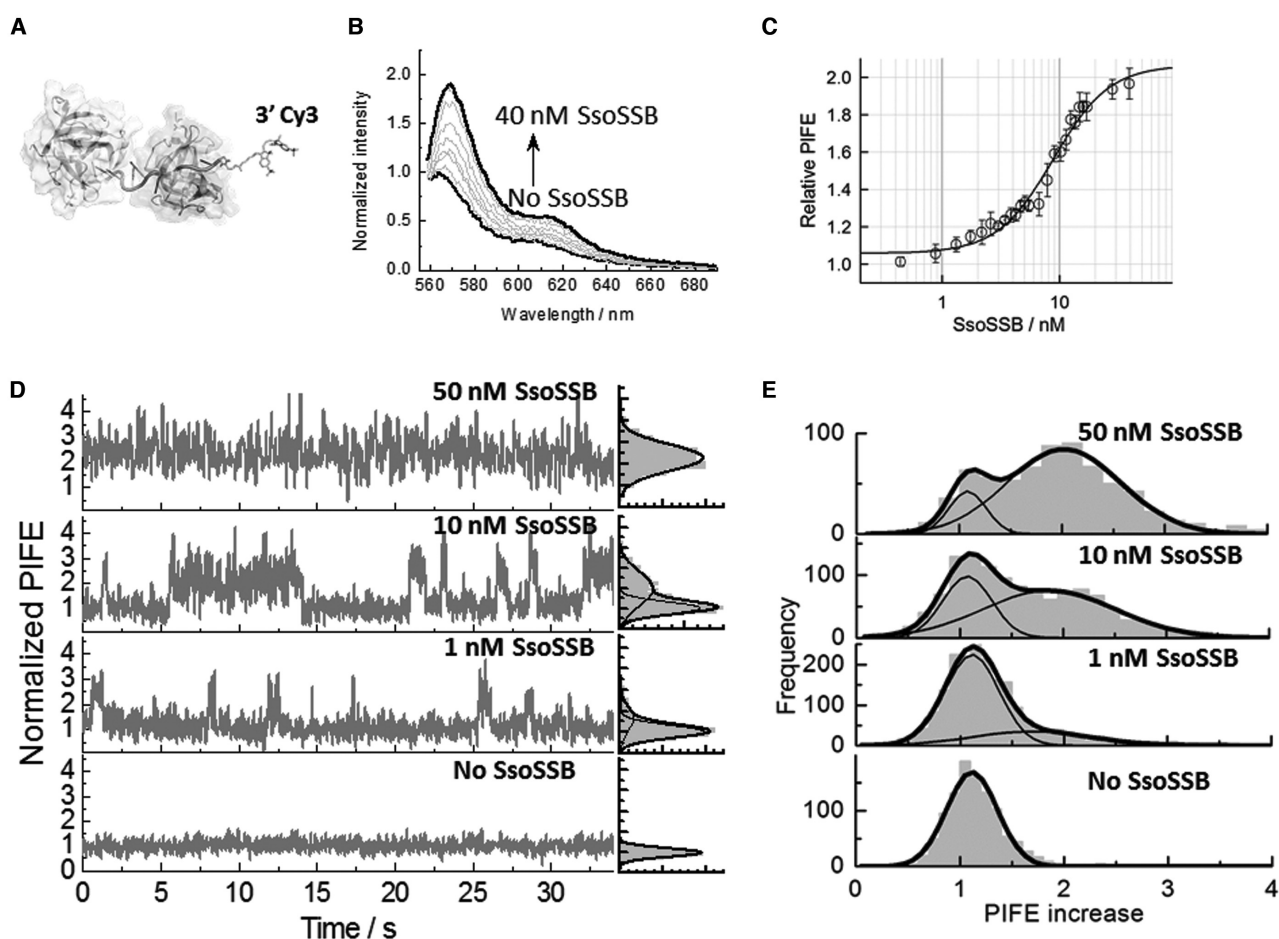
SsoSSB binding to a 12-mer single-strand DNA monitored by protein-induced fluorescence enhancement (PIFE). (**A**) Molecular modelling of SsoSSB monomers (see Supplementary section for details) bound to a 12-mer Cy3-labelled single strand. (**B**) Fluorescence emission spectra of the Cy3 fluorophore inserted at the 3′ termini of a 12-mer dC sequence as a function of SsoSSB concentration. The fluorescence spectrum in the absence of SsoSSB was normalized to unity at the wavelength of the maximum and taken as a reference to calculate the relative increase in emission intensity at each SsoSSB concentration. (**C**) Relative variation in emission intensity of Cy3 normalized with respect to the emission intensity obtained in the absence of SsoSSB in a background of 10 mM KCl. Values represent the average of three experiments and are given as mean ± s.e.m. Solid line indicates the non-linear squares fit to Equation S1 as described in the Supplementary section. *K*_D_ and Hill coefficient values of 9 ± 1 nM and 1.8 ± 0.2 were obtained, respectively. (**D**) Representative single-molecule trajectories of Cy3 emission intensity obtained at the indicated concentrations of SsoSSB with 50 ms integration time. SsoSSB association and dissociation can be observed as PIFE events in the single molecule trace. The raw Cy3 intensity in the absence of DNA has been normalized to unity and this has been taken as the signal reference for the single-molecule trajectories to quantify the relative increase in Cy3 intensity due to SsoSSB binding. Corresponding single-molecule PIFE histograms for each trace are also shown on the right panels. (**E**) Cumulative single-molecule histograms and Gaussian fitting (solid lines) of normalized Cy3 intensity built from >1000 molecules at each concentration of SsoSSB.

Here, we employed PIFE to monitor the interaction between SsoSSB and a 12-mer dC single-strand sequence labelled at the 3′ position with Cy3, referred to hereafter as (dC_12_Cy3). We have chosen a 12-mer ssDNA because it is well within the distance range of PIFE and therefore an increase in the emission of Cy3 is expected upon SsoSSB binding (Figure 1A). To demonstrate this in bulk solution, we performed an equilibrium titration of dC_12_Cy3 with SsoSSB at 10 mM KCl and 20°C (Figure 1B). Although the majority of biophysical studies characterizing SSB proteins, particularly at single-molecule level, have been carried out at room temperature (44), even for thermophilic species such as Thermus SSB (36); we also carried out a PIFE titration at 65°C (Supplementary Figure S1), which is closer to the optimal growth temperature of *S. solfataricus* (75–80°C). As we increased the concentration of SsoSSB, we observed, at both temperatures, a similar 2-fold increase in the emission intensity of the Cy3 spectra due to SsoSSB binding in close proximity to the dye. The binding isotherm in Figure 1C was fitted to a Hill binding model (Equation S1) (45) yielding a dissociation constant (*K*_D_) of 9 ± 1 nM, which is very good agreement with values previously reported at room temperature for SsoSSB (32) and archaeal RPAs (46). This confirms also that the presence of the Cy3 label at the 3′ end of dC_12_Cy3 does not significantly affect SsoSSB binding. From the fitting we obtained a Hill coefficient of 1.8 ± 0.2, implying the interaction of more than one protein with the ssDNA with a significant degree of positive cooperativity between them. Similar positive cooperativity with Hill coefficient values close to the one reported here have been described for the oligomeric RPA1 from *Methanosarcina acetivorans* (47). Fitting the binding isotherm obtained at 65°C yielded <2-fold increase in the dissociation constant (15 ± 1 nM) and a Hill coefficient value (1.4 ± 0.2) that suggest a certain degree of cooperativity operating also at high temperatures. Based on the similar behaviour obtained at both temperatures, we decided to continue the characterization of SsoSSB exclusively at room temperature.

We next used a prism-based TIR microscopy approach to gain further insights into the binding dynamics of SsoSSB to dC_12_Cy3 at single-molecule level using PIFE. For this, we introduced a biotin moiety to the 5′ end of the single strand DNA (BidC_12_Cy3) to act as an anchoring point to streptavidin-coated quartz slides. Intensity traces of surface-immobilized Cy3-labelled DNA were initially measured in the absence of SsoSSB (Figure 1D). At these conditions, the majority of traces (>90%) showed Cy3-intensity levels that were stable over relatively long periods of time (∼1 min) and the cumulative single-molecule histogram displayed a single Gaussian with a narrow width distribution (Figure 1E). In addition to confirming that DNA alone does not exhibit signal fluctuations, these data were taken as a reference to quantify the magnitude of the PIFE effect induced by protein binding. Upon addition of 1 nM SsoSSB, the fluorescence signal normalized to the lower intensity value of the unbound state showed dynamic fluctuations to a Cy3 emissive state with a ∼2-fold higher brightness (Figure 1d and Supplementary Figure S2a).

A higher frequency of fluctuations between these two states was observed when the concentration of protein was increased to 10 nM, suggesting that they arise from binding and dissociation events of SsoSSB proteins (Figure 1D and Supplementary Figure S2b). To investigate whether these events were due to monomers or multimeric species of SsoSSB present in solution we carried out a range of fluorescence assays using either mixtures of labelled and unlabelled SsosSSB or only labelled SsoSSB. We did not observe any variation in fluorescence anisotropy when A647-SsoSSB was titrated with unlabelled protein and we did not detect any FRET signal between a mixture of A647-SsoSSB and Cy3-SsoSSB. To ensure that the absence of interaction was not due to the relative low concentration of SsoSSB required in fluorescence methods, we developed an electron paramagnetic resonance (EPR) assay using SsoSSB carrying a 1-Oxyl-2,2,5,5-tetramethylpyrroline-3-methyl) spin label at the same position as the fluorescent dye (see ‘Materials and Methods’ section for details). The EPR experiments using up to 100 μM of spin labelled SsoSSB failed to provide any evidence for multimeric species formed in solution as discussed later in this section (Supplementary Figure S3). Thus, we concluded that the observed PIFE events arise from the association and dissociation dynamics of individual SsoSSB monomers to the ssDNA.

At 50 nM concentration of SsoSSB, the normalized intensity traces displayed a single emission level with a constant 2-fold enhancement (Figure 1D). We interpreted this as evidence for the BidC_12_Cy3 strand becoming completely coated with SsoSSB monomers. In contrast to the behaviour observed for RecA binding to ssDNA (42), no discrete fluctuations between two emission levels, caused by the stepwise association of one or two monomers to the BidC_12_Cy3 strand, were detected at any concentration of SsoSSB. This suggests that either the binding dynamics of two monomers is faster than our time resolution (33 ms), or the binding of a second monomer of SsoSSB to the BidC_12_Cy3/(SsoSSB) complex induces a minimal change in the micro-viscosity of the medium surrounding the Cy3 dye. The normalized cumulative histograms for more than 1000 molecules obtained at each concentration of SsoSSB confirmed the behaviour observed from individual intensity traces (Figure 1E). The relative contribution of the single-molecule histogram representing a 2-fold enhancement in Cy3 intensity increased as a function of SsoSSB concentration, reaching a value of 86% of the total at 50 nM SsoSSB. Interestingly, a comparison of the Gaussian distributions corresponding to the populations of unbound and bound ssDNA revealed a remarkable increase in their full width at half maximum (FWHM). The FWHM changed from a value of 0.4 for BidC_12_Cy3 alone (Figure 1E, bottom panel) to 1.1 for the histogram of the BidC_12_Cy3/(SsoSSB)_2_ complex centred at a PIFE value of ∼2 (Figure 1E, top panel). In addition to the intrinsic flexibility of the ssDNA, we speculate that such increase might also reflect a loose organization of the SsoSSB monomers on the BidC_12_Cy3 strand, with some arrangements inducing a substantially higher increase in local viscosity. To investigate this further, we employed site-directed mutagenesis to generate and purify a SsoSSB variant (A114C) and labelled this cysteine residue with the spin label MTSSL for pulsed electron-electron double resonance (PELDOR) measurements (Supplementary section) (48, 49). The MTSSL-labelled SsoSSB did not produce any PELDOR modulation in the absence of ssDNA, even at a protein concentration of 100 μM (Supplementary Figure S3). The analysis of the modulation depth of the EPR signal (50) indicates that ∼90% of the SsoSSB was monomeric in the absence of DNA. The increase in the modulation depth and the broad distribution of distances observed upon addition of 50 μM ssDNA (Supplementary Figure S3) supports a model where up to ∼40% of SsoSSB monomers associate next to each other only when they associate on the same ssDNA.

### Adjacent binding of dye-labelled SsoSSB monomers to short ssDNA fragments results in complete quenching of the fluorescence emission

**Figure 2.**
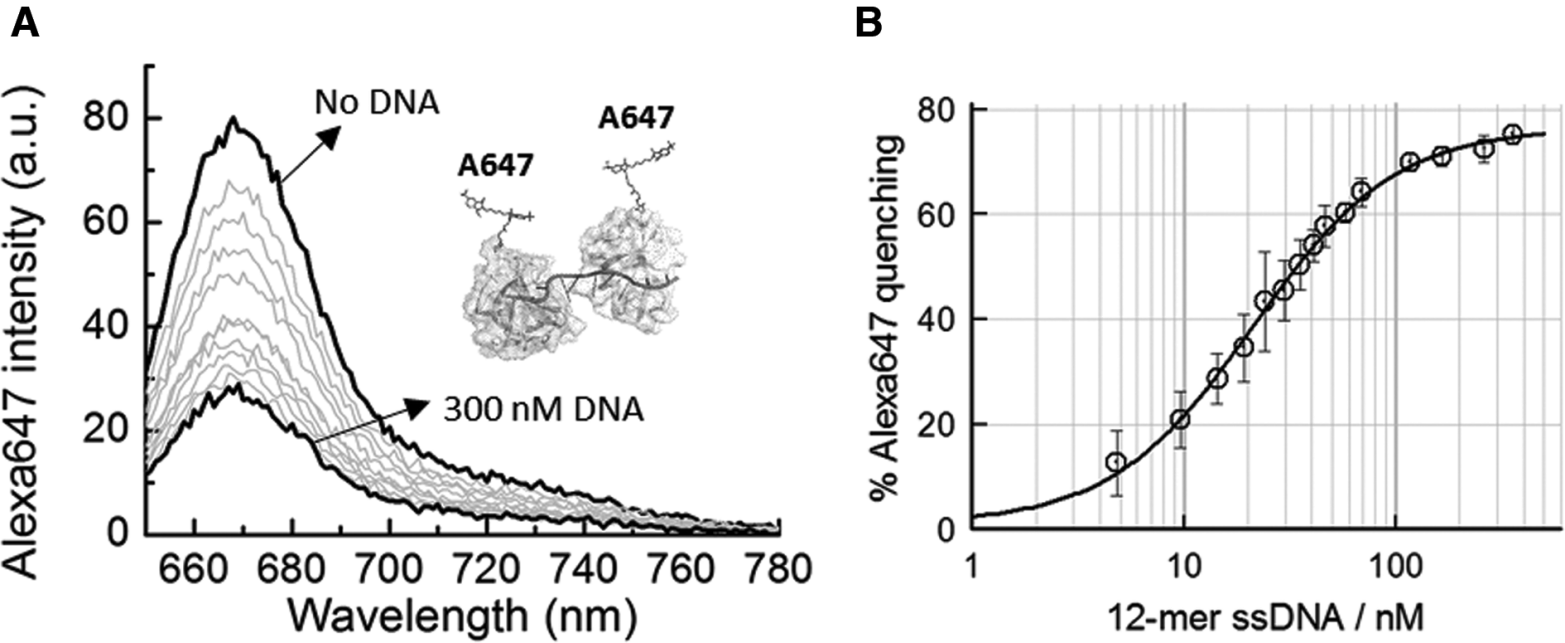
Fluorescence quenching as a reporter of adjacent binding between Alexa647-labelled ssoSSB monomers. (**A**) Fluorescence spectra of Alexa647-labelled SsoSSB as function of increasing concentrations of a 12-mer dC single-stranded DNA. (**B**) Percentage of quenching of Alexa647 intensity as a function of ssDNA concentration in a background of 10 mM KCl. Values represent the average of three experiments and are given as mean ± s.e.m. Solid line indicates the non-linear squares fit to Equation S1 as described in the Supplementary section. *K*_D_ and Hill coefficient values of 20 ± 1 nM and 1.3 ± 0.2 were obtained, respectively.

To probe the binding mechanism in further detail, we decided to explore the use of dye-labelled SsoSSB proteins to monitor the stepwise association of SsoSSB monomers to surface-immobilized unlabelled ssDNA substrates. The A114C SsoSSB variant was labelled at this cysteine residue with Alexa Fluor 647 (A647) (Supplementary section) and an ensemble titration of A647-SsoSSB at increasing concentrations of the dC_12_ strand was carried out. As shown in Figure 2A, we observed a progressive decrease in the quantum yield of Alexa Fluor 647 upon addition of ssDNA, reaching an 80% quenching value at 300 nM concentration of SsoSSB. We interpreted this as evidence that the adjacent assembly of A647-labelled SsoSSB monomers in the dC_12_ strand brings the Alexa Fluor 647 fluorophores into close proximity to favour a self-quenching mechanism. Self-quenching has been already used to monitor the formation of aggregates between Cy3-labelled peptides (51) and self-quenching between tetramethylrhodamine (TMR) dyes has also been used in ensemble and single-molecule studies to monitor intramolecular conformational changes in proteins and nucleic acids (52). The binding isotherm for A647-SsoSSB was fitted to the Hill binding model described by Equation S1 (Figure 2B). We obtained a dissociation constant of 22 ± 1 nM and a Hill coefficient of 1.4 ± 0.2, suggesting again that the adjacent binding of two SsoSSB monomers exhibits a significant degree of positive cooperativity.

To test that the observed quenching was indeed caused by the adjacent binding of two SsoSSB monomers on the ssDNA strand, we repeated the titration of A647-SsoSSB, but this time, replacing the dC_12_ strand by a dT_6_ strand that can only accommodate a single monomer. No significant quenching was observed for dT_6_ even when the concentration of SsoSSB was increased well beyond that previously employed to titrate the dC_12_ strand (Supplementary Figure S4a and b). To rule out the possibility that the lack of quenching was due to a compromised binding affinity for short ssDNA fragments, we carried out an experiment using the intrinsic fluorescence of tryptophan as a reporter of binding. As shown in the crystal structure, SsoSSB contains two tryptophan residues in the OB-fold domain, Trp56 and Trp75, that are important for ssDNA binding (24). Fluorescence titration of these tryptophan residues has been previously used to report the binding affinities of wild-type SsoSSB for different lengths of ssDNA (24, 32). In our case, when a solution of wild-type SsoSSB was titrated with 6-mer DNA, in a similar range of concentrations as those used for A647-SsoSSB, we observed a progressive quenching of the tryptophan emission upon excitation at 290 nm (Supplementary Figure S5a and b). The binding isotherm was fitted to a Hill model as described by Equation S1 (Supplementary section) to yield a dissociation constant of 143 ± 95 nM (Supplementary Figure S5b), indicating that SsoSSB can indeed bind the 6-mer ssDNA fragment although with a lower affinity than to a 12-mer fragment. We speculate that this decrease in affinity might be due to the instability of the complex formed in a fragment whose length is near the footprint of the SsoSSB. We also tested whether the observed quenching was caused by the dye present on the adjacent protein or by the protein itself. For this, we carried out a similar titration as that shown in Figure 2 but using a mixture of A647-SsoSSB and unlabelled protein in a ratio 1:10, respectively. At these conditions we only observed 12% quenching of the emission of the Alexa Fluor 647 dye (Supplementary Figure S6). Overall, these data confirm the presence of an efficient self-quenching mechanism of Alexa Fluor 647 dyes located in adjacent SsoSSB monomers, a feature that we have exploited later in this work to interpret the SsoSSB binding dynamics at single-molecule level.

### Intermolecular ensemble-FRET characterization of SsoSSB interaction with single-strand DNA

In the previous sections, we demonstrated that the association of SsoSSB with the ssDNA strand influences the emission properties of Cy3 labelled ssDNA and A647-labelled SsoSSB and leads to the appearance of PIFE and quenching processes in their respective complexes with unlabelled partners. Using this knowledge and the fact that Cy3 and Alexa Fluor 647 dyes constitute a donor/acceptor pair commonly used in FRET assays (37, 41), we combined both fluorescent labels within the same complex to generate an inter-molecular FRET assay that can provide complementary information to that obtained by PIFE and quenching. In the context of SSB proteins, FRET-based assays have been crucial to elaborate a molecular level understanding of *Eco*SSB (34–36) and human RPA function (53), the dynamics of filament formation by RecA (54) and the organization of muti-protein complexes on DNA substrates (43, 55).

**Figure 3.**
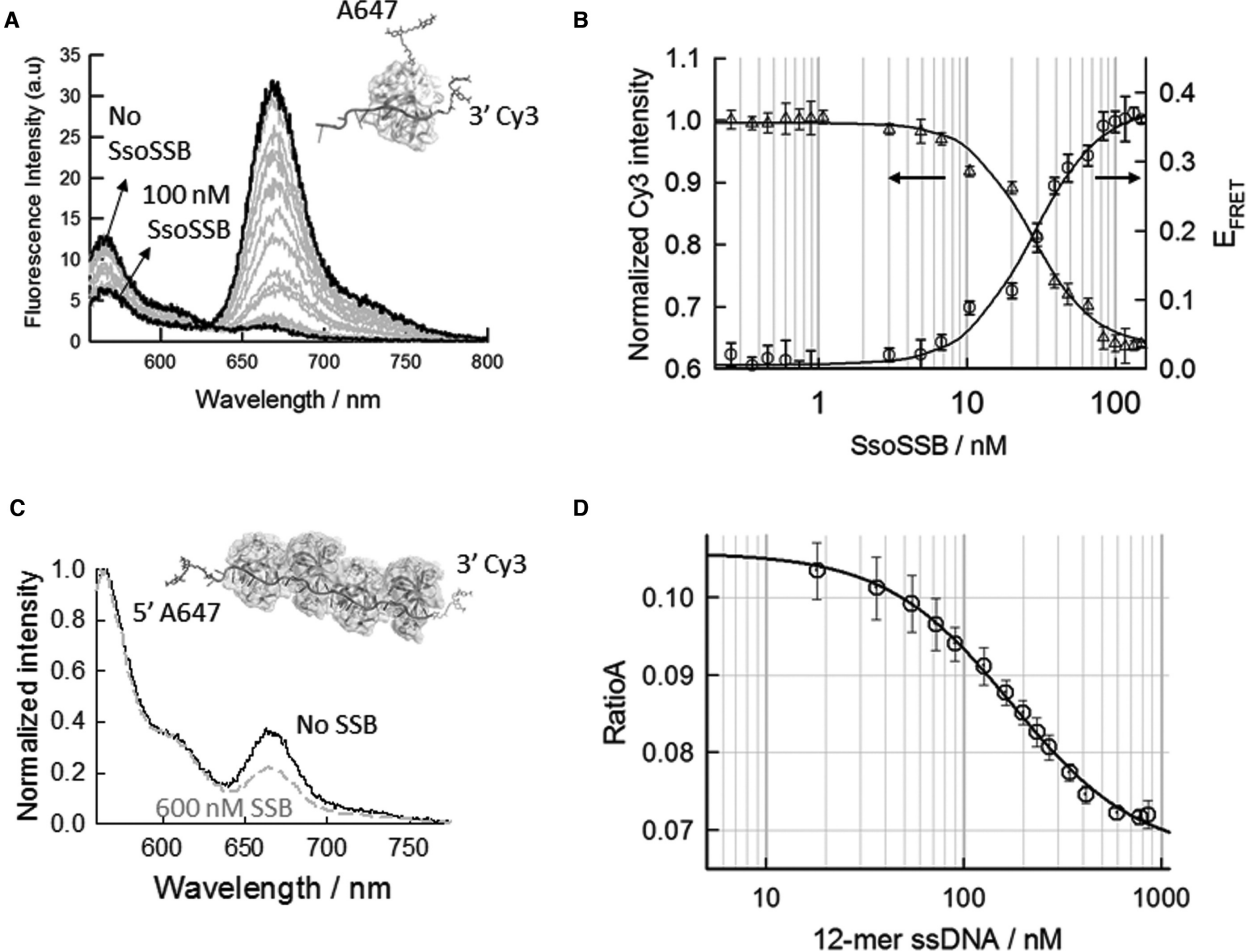
SsoSSB binding to ssDNA and associated conformational changes monitored by intra- and intermolecular FRET. (**A**) Intermolecular FRET assay to monitor Alexa647-labelled SsoSSB binding to 12-mer dC ssDNA strand labelled with the FRET donor Cy3. The fluorescence spectra of Cy3 and Alexa647 was collected in the range 555–800 nm upon excitation of the donor at 547 nm as function of increasing concentrations of Alexa647-SsoSSB. (**B**) FRET binding isotherm and normalized Cy3 donor intensity as a function of SsoSSB concentration. Plotted values represent the average of three experiments and are given as mean ± s.e.m. Solid lines represent the non-linear squares fit to a Hill model (see Supplementary section for details). (**C**) Intramolecular FRET assay to monitor conformational changes in a 39-mer dC ssDNA induced by SsoSSB binding. Fluorescence spectra of Cy3 and Alexa647 normalized at the maximum of the Cy3 emission band (565 nm) in the absence and presence of 600 nM SsoSSB are shown. (**D**) Variation in the RatioA value as a function of SsoSSB concentration. RatioA values were calculated as described in the Supplementary section and represent the average of three experiments. The solid lines indicates the results from a non-linear squares fit to Equation S1 as described in the Supplementary section. *K*_D_ and Hill coefficient values of 174 ± 20 nM and 1.4 ± 0.2 were obtained, respectively.

We therefore re-examined the binding properties of A647-SsoSSB by ensemble-FRET using similar experimental conditions and the same DNA strand, dC_12_Cy3, used for the PIFE studies (Figure 1B and C). In the absence of A647-SsoSSB, the fluorescence emission obtained with excitation at the maximum of the donor absorption band (547 nm) showed the characteristic band of the Cy3 donor centred at 560 nm (Figure 3A). As the concentration of A647-SsoSSB protein was increased, we observed a progressive decrease in the donor emission band and a concomitant increase in the emission band of the acceptor species centred at 660 nm (Figure 3A). We assigned this as evidence of the donor and acceptor species coming into close proximity and triggering an efficient energy transfer process between the two partners within the complex. The efficiency of inter-molecular energy transfer as a function of added A647-SsoSSB was evaluated by quantifying the decrease in the donor intensity using Equation S2 (Supplementary section) and fitting the corresponding binding isotherm to the Hill binding model (Figure 3B). We obtained values for the dissociation constant and the Hill coefficient of 24 ± 2 nM and 1.8 ± 0.2, respectively. These values are in good agreement with those previously obtained when using PIFE and acceptor quenching (Figure 1B and C).

Surprisingly, despite the close positioning of the FRET pair that could be expected on the 12-mer strand, the FRET efficiency increased only to a moderate value of E∼0.35 (Figure 3B). To understand the reason for this low FRET efficiency, we modelled the dC_12_Cy3/(Alexa647SsoSSB)_2_ complex taking into account the position of the dyes and using the crystallographic structure of the RPA70:ssDNA complex as a template (see details in Supplementary section). Although the model constitutes only a simplified approximation to the structure of the complex in solution, it allowed us to confirm that an A647-SsoSSB binding near the Cy3 on the ssDNA results in an approximate average inter-dye distance of 35–37 Å (E_FRET_ ∼ 0.8–0.9) (Supplementary Figure S7), whereas binding near the unlabelled end of the ssDNA yields a distance ranging from 65 Å to 69 Å (E_FRE__T_ ∼ 0.28–0.36), depending on the relative orientation of the SsoSSB on the ssDNA (Supplementary Figure S7). From this, it is clear that a random distribution of one monomer or two monomers bound to the ssDNA should result in a higher FRET efficiency than that experimentally obtained (E_FRET_ ∼ 0.35). In fact, if we take into account that the increase in quantum yield of the Cy3 donor due to PIFE (Figure 1B and C) will further increase the Förster distance (R_0_), we will expect an experimental FRET efficiency higher than that predicted assuming a constant R_0_ ∼ 60 Å. From the experiments using Alexa Fluor 647 and unlabelled ssDNA described before, we know the adjacent binding of two monomers induces a pronounced quenching of the Alexa Fluor 647 dye. Therefore, the low FRET values observed can be interpreted assuming the stepwise binding of one monomer leading to FRET, followed by a second monomer that leads to acceptor quenching and places the acceptor in a FRET-inactive state. This scenario is reminiscent of the well-known transient blinking of cyanine dyes and derivatives generated by transitions to FRET inactive states including triplet and radical anionic energy levels (56). Although the exact nature of the Alexa Fluor 647 inactive state in the complex dC_12_Cy3/(Alexa647)_2_ requires further investigation, the variation in E_app_ and Cy3 intensity was well fitted assuming a two-site model (Equation S3) that accounts for the stepwise association of two monomers to the ssDNA. We obtained values of 37 ± 4 nM and 2.8 ± 1 nM, for the binding of the first and the second monomer, respectively. As previously reported using the quenching and PIFE assays, these results confirm a certain degree of positive cooperativity for the binding of the second monomer to the 12-mer DNA.

### Conformational changes in ssDNA induced by SsoSSB binding

Ensemble-FRET has also been extensively used to investigate the structural distortion of the ssDNA strand upon binding to SSBs containing multiple OB-folds (34–36). For this, the variation in the end-to-end distance of ssDNA between unbound and SSB-bound ssDNA has been taken as a parameter to determine the protein binding mode, but such analysis is still lacking for SsoSSB. Thus, to evaluate the organization of SsoSSB proteins coating a ssDNA strand, we have engineered a 39-mer ssDNA labelled at the 3′- and 5′-termini with Cy3 and Cy5 (Cy5dC_39_Cy3), respectively. By positioning the Cy3-Cy5 FRET pair at both ends of the ssDNA, variations in the efficiency of the FRET process as a function of added protein provide a measure of relative changes in the conformation of the ssDNA induced by SsoSSB. The 39-mer ssDNA was also chosen to ensure a sufficiently long DNA fragment so that any potential multimeric species that could wrap the ssDNA could be stably formed, as observed for EcoSSB (34, 36).

In the absence of SsoSSB, the fluorescence spectrum of Cy5dC_39_Cy3 in a background of 10 mM KCl shows a predominant band at short wavelength due to the Cy3 donor and a much smaller peak corresponding to the sensitized emission of the Cy5 acceptor (Figure 3C). Using the RatioA method (41) (Supplementary section), we calculated a FRET efficiency (E_FRET_) of ∼0.08, which is very close to the value of 0.1 reported for a Cy5dT_40_Cy3 strand at 20 mM KCl (57). A slightly lower value for the FRET efficiency at lower ionic strength is justified on the basis of well-known influence of mono- and divalent metal ions screening the negative changes on the nucleic acid backbone. Using a Förster distance (R_0_) of 60 Å for the Cy3/Cy5 pair, as reported in the literature (37, 41), a value of E_FRET_ ∼ 0.08 corresponds to an inter-dye distance (R) of ∼90 Å. Upon addition of increasing concentrations of SsoSSB, the FRET efficiency progressively decreased as reflected by the relative decrease in the emission of the Cy5 band with respect to the Cy3 peak (Figure 3c), and reached a saturation value of E_FRET_ ∼ 0.02 at 780 nM SsoSSB and 10 mM KCl (Figure 3D). This corresponds to an inter-dye distance of R ∼ 114 Å for the SsoSSB coated Cy5dC_39_Cy3 strand. This increase in end-to-end distance is in contrast to the behaviour observed for the different binding modes of SSBs such as *E. coli* (22) and RPA (20, 21), where wrapping the ssDNA around the multimeric structure results in a shortening of the dye-to-dye distance and an increase in FRET efficiency.

### Dissecting the interaction between SsoSSB and ssDNA using a combination of quenching, PIFE and FRET single-molecule events

**Figure 4.**
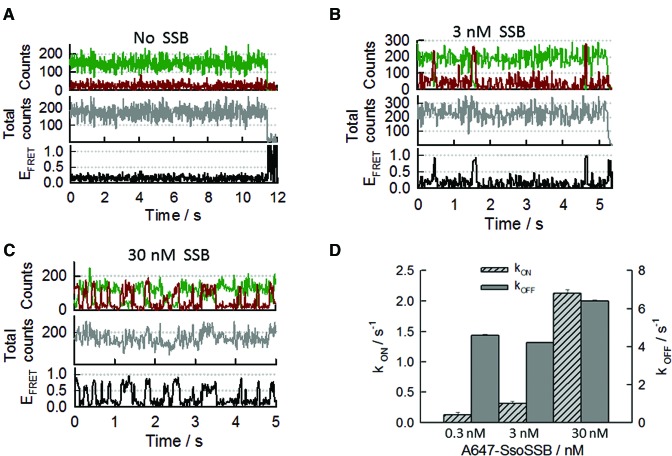
Real-time single-molecule intermolecular FRET measurements of SsoSSB binding to a 20-mer ssDNA (BidC_20_Cy3). The DNA was labelled at the 3′ terminus with the Cy3 donor and immobilized on the quartz slide using biotin-streptavidin interactions. SsoSSB was labelled with Alexa647 as FRET acceptor (**A**) Single-molecule donor and acceptor trajectory (upper panel), total intensity (middle panel) and FRET trace (bottom panel) obtained in the absence of SsoSSB. Photobleaching of the Cy3 donor occurred at ∼11 s. (**B**) Representative single-molecule binding dynamics of Alexa647-SsoSSB to the BidC_20_Cy3 ssDNA. Anti-correlated fluctuations in the donorand acceptoremission signals (upper panel) are indicative of SsoSSB association and dissociation events. The corresponding PIFE and FRET trajectories are shown in the middle and bottom panel, respectively. (**C**) Representative single-molecule donor and acceptorintensity trajectory showing the binding dynamics of SsoSSB to BidC_20_Cy3 obtained at 30 nM concentration of Alexa647-labelled SsoSSB (upper panel). PIFE trajectory (middle panel) and FRET trace (bottom panel) are also shown. (**D**) Bar plot showing the association (*k*_ON_) and dissociation (*k*_OFF_) rates in s^−1^ obtained for Alexa647-SsoSSB binding to BidC_20_Cy3 at the indicated concentrations of SsoSSB. Rates and associated errors were obtained from the fitting of the single-molecule dwell-time histograms to a mono-exponential decay function.

We next examined by single-molecule FRET the binding dynamics of A647-SsoSSB to a 20-mer ssDNA labelled at the 3′-end with Cy3 using a TIR microscope. To allow immobilization of the ssDNA on the quartz slide, we introduced an additional biotin group at the 5′ end of the ssDNA (BidC_20_Cy3). In the absence of SsoSSB, the single-molecule trajectories displayed stable Cy3-intensity signals until photobleaching occurred (Figure 4A) and we calculated a Cy3 lifetime before photobleaching of ∼55 s (*k*_bleaching_ = 0.018 ± 0.002 s^−1^) (Supplementary Figure S8) at 200 W/cm^2^ of excitation power. This value for the photobleaching rate is very similar to that reported in the literature using the same oxygen scavenger system (58). Upon addition of a 3 nM concentration of A647-SsoSSB, the trajectories displayed short-lived anti-correlated transitions between the intensity signals of the donor and acceptor fluorophores, whilst the total intensity remained constant across the entire trajectory (Figure 4B and Supplementary Figure S9a). The single-molecule FRET traces obtained from the corresponding intensity signals (see details in Supplementary Materials and Methods) showed rare transitions from a very low FRET value (E_app_ ∼ 0.1), which we assigned to the unbound ssDNA state, to a very high value (E_app_ ∼ 0.8–0.9) representing the binding of A647-SsoSSB to the BidC_20_Cy3 strand that brings the FRET pair in close proximity. This assignment is supported by the increase in the population of the high-FRET state observed when the concentration of SsoSSB increased to 30 nM (Figure 4C and Supplementary Figure S9b). From the analysis of the dwell times of the low- and high-FRET states, we extracted the association and dissociation rates at each concentration of SsoSSB investigated (Figure 4D). The binding rate showed a 16-fold increase from a value of 0.13 ± 0.04 s^−1^ at 0.3 nM to 2.14 ± 0.06 s^−1^ at 30 nM concentration, whereas the dissociation rate remained mostly constant within the error with values ranging from 4.52 ± 0.03 to 6.34 ± 0.01 s^−1^. To confirm that the observed transitions from high to low FRET were due to dissociation events and not caused by Alexa Fluor 647 photobleaching, we calculated the photobleaching rate of A647-SsoSSB monomers. For this we used the extrusion method to encapsulated the protein within a 100 nm diameter eggPC vesicle (40). The vesicle was then immobilized in a quartz slide to directly image the dye using a 633 nm excitation wavelength. We obtained an average lifetime of A647-SsoSSB of ∼15 s before photobleaching (*k*_bleaching_ = 0.07 ± 0.01 s^−1^) (Supplementary Figure S8). This value is ∼60× slower than the dissociation rate of a single A647-SsoSSB monomer from ssDNA (4.52 ± 0.03 s^−1^) and confirmed our assignation of the transitions as resulting from binding dynamics and not dye photophysics.

**Figure 5.**
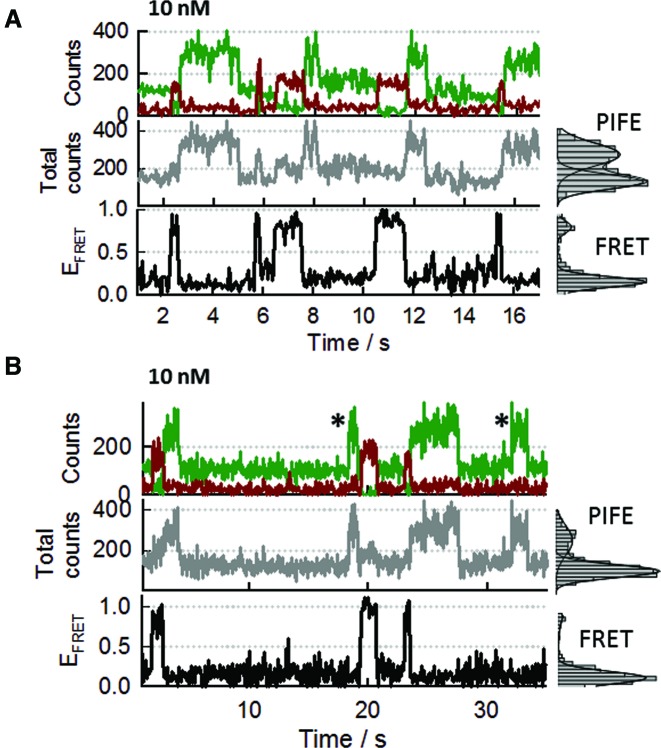
SsoSSB binding to a short ssDNA segment leads to single-molecule trajectories showing coexisting FRET, PIFE and acceptor quenching events. (**A**) Representative single-molecule trajectory showing the effect that SsoSSB binding to a surface-immobilized 12-mer ssDNA labelled at the 3′ end with Cy3 (BidC_12_Cy3) has on the donor and acceptor intensities (upper panel), the PIFE signal (middle panel) and the FRET efficiency (bottom panel). Panels on the right show the single-molecule histograms for the corresponding trajectory displayed on the left. (**B**) Single-molecule trajectories and corresponding histograms obtained at identical conditions as those described for (A) but showing additional rare events (marked with an asterisk). As shown in panel (A), the majority of PIFE events formed quickly following a high-FRET state and only a small percentage (panel B, marked with asterisk) was observed to directly emerge from a non-FRET state (no protein bound).

Concentrations higher than ∼30 nM of A647-SsoSSB that would enhance the probability of SsoSSB monomers associating at adjacent sites on the ssDNA, would also increase the background signal and compromise the spatial separation between immobilized species that is essential for single-molecule experiments. Therefore, we decided to reduce the length of the ssDNA to 12-mer (BidC_12_Cy3) to encourage such events at concentrations of A647-SsoSSB still compatible with single-molecule imaging. Representative single-molecule trajectories obtained for the binding of 10 nM A647-SsoSSB to BidC_12_Cy3 are shown in Figure 5a and b. In addition to anticorrelated fluctuations between donor and acceptor intensities (Figure 5a and b, upper panel), reflecting variations in FRET efficiency (bottom panel), we also observed variations in donor and total intensity signals (Figure 5A and B, middle panel) that most likely arise from quenching and PIFE events. A comparison of the single-molecule trajectories obtained at 10 nM SsoSSB and 150 mM KCl revealed a lower binding frequency and a substantial reduction in the formation of S4 states (Supplementary Figure S10). Although an analysis of the influence of ionic strength on the binding dynamics of SsoSSB requires a more detailed investigation, a similar effect has been observed previously for other multimeric SSBs and has been interpreted on the basis of a decrease in the binding affinity for ssDNA at high ionic strength (4, 16, 19, 34, 36).

**Figure 6.**
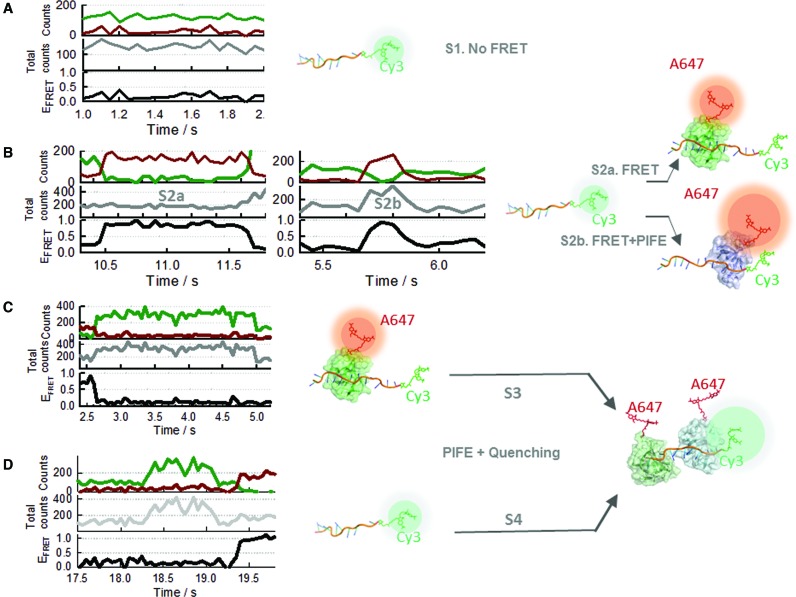
Dissecting the binding mechanism of SsoSSB to ssDNA using a single-molecule multi-process approach. Each single-molecule event (FRET, PIFE and acceptor-quenching) observed in the binding trajectories was assigned to an specific interaction between SsoSSB and the 12–mer ssDNA as described in panels (**A**–**D**). (A) *S1-state:* no-FRET state corresponding to unbound Cy3-labelled DNA. Single molecule trajectories displayed emission only from the donor (green) (left panel). (B) *S2-state:* high-FRET state formed by monomer binding to the ssDNA resulting in SsoSSB:ssDNA complexes of 1:1 stoichiometry. This state is characterized by an anti-correlated transition between donor (green) and acceptor (dark red) emission. Depending on the relative positioning of the bound monomer with respect to the Cy3, the total intensity might remain constant (*S2a*) or exhibit an increase due to PIFE (*S2b*). (C and D) Formation of ssDNA:(SsoSSB)_2_ complexes takes place by incorporation of a second monomer to a previously formed SsoSSB:DNA complex (pathway S3). This pathway is characterized by the transition from a high-FRET state (S2 state) to a no-FRET state where the Cy3 intensity, and the total intensity, has increased by 2-fold due to PIFE, but there is no emission from Alexa647 because of quenching between adjacent SsoSSB monomers. Some events leading to the formation of ssDNA:(SsoSSB)_2_ complexes (pathway S4) are faster than the time resolution of our EMCCD camera (33 ms) and appear as a single-step transition involving a PIFE-induced increase in Cy3 and total intensity emission (panel D).

Through the analysis of 500 single-molecule trajectories at 10 nM SsoSSB and 10 mM KCl, we identified four distinct single-molecule behaviours as shown from Figure 6A–D. The first state, S1, represents ssDNA with no SsoSSB bound and it is characterized by the presence of intensity only from Cy3 and no FRET signal (Figure 6A). The second group, S2a includes anticorrelated fluctuations between the donor (Cy3) and acceptor (Alexa Fluor 647) signals (Figure 6B, left panel) with the total emission intensity remaining constant (Figure 6B, middle panel). This corresponds to FRET events as usually observed in single-molecule FRET, and in the context of SsoSSB binding, they can be assigned to monomers interacting with the ssDNA at positions relatively distant to the Cy3 location for PIFE to have a significant impact. This differs from the behaviour reported in the middle panel of Figure 6B (state S2b), where the anticorrelated fluctuation of the Cy3 and Alexa Fluor 647 intensities is accompanied by a concomitant increase in the total intensity for the same time period. We reasoned that these S2b states arise from protein binding events taking place at PIFE-distance from the Cy3 donor. The proximity of the protein induces an increase in the quantum yield of the Cy3 dye by PIFE, resulting in a faster rate of energy transfer (higher R_0_) and a more efficient population of the acceptor (55, 59). To investigate if the proximity of the bound monomer to the Cy3 dye was responsible for the difference between the S2a and S2b states, we increased the ionic strength to 150 mM KCl. At these conditions, the fragment of the ssDNA that was not occluded by the bound monomer should adopt a collapsed conformation, thus placing the dye in close proximity to the SsoSSB and potentially allowing a more efficient PIFE process. Representative traces obtained at these conditions are shown in Supplementary Figure S10. We observed that the majority of high-FRET states showed a high Cy5 emission which is characteristic of S2b states involving very efficient FRET and PIFE processes. This result confirms that the formation of S2b states only takes place from SsoSSB monomers binding near the Cy3 or when the ssDNA is able to adopt a collapsed state. We conclude that the PIFE mechanism induced by SsoSSB requires a much closer positioning to the fluorophore than that observed for other proteins such as RecA (42).

The S3 state includes single-molecule states showing no emission from the acceptor but with the Cy3 emission level and the total intensity displaying a ∼2-fold increase with respect to the unbound state (Figure 6C). We assigned this state to the formation of BidC_12_Cy3/(A647-SsoSSB)_2_ complexes. In this complex, the proximity of SsoSSB to the donor increases its quantum yield by PIFE and the presence of two SsoSSB monomers adjacent to each other quenches the emission of the Alexa Fluor 647 dye, as shown by ensemble methods.

The last state, S4, corresponds to donor and acceptor intensity levels similar to those described for state S3, but the formation of this state always takes place from S1 (no SsoSSB bound) instead of S2 (Figure 6D, left panel). The observation of direct S1→S4 transitions could be explained either by considering the association to the ssDNA of A647-SsoSSB dimers pre-formed in solution or if the second monomer binds to the ssDNA at a faster rate than our time-resolution (33 ms), or a combination of both. The frequency of the S1→S4 transitions changed very little with the concentration of SsoSSB and reached a maximum contribution of ∼17% of the total number of transitions observed at the highest concentration of SsoSSB investigated. Our single-molecule data do not allow us to assign this contribution to a small fraction of dimers formed in solution or to the fast association of a second monomer. However, the lack of EPR modulation depth observed in the absence of ssDNA, even at concentrations of SsoSSB in the micromolar region (Supplementary Figure S3), led us to conclude that S1→S4 transitions most likely arise from the association of the second monomer at a rate beyond our time resolution. Overall, FRET and PIFE have been previously employed in separate single-molecule experiments and also within the same trajectory to characterize protein–DNA interactions (42, 43). However, our data constitute the first example of using the direct competition between FRET, PIFE and quenching within each trajectory to reveal dynamic information that otherwise will be inaccessible; a feature that might be useful for the analysis of other complex DNA–protein interactions.

### Monomeric SsoSSB is the functional unit recognizing ssDNA

**Figure 7.**
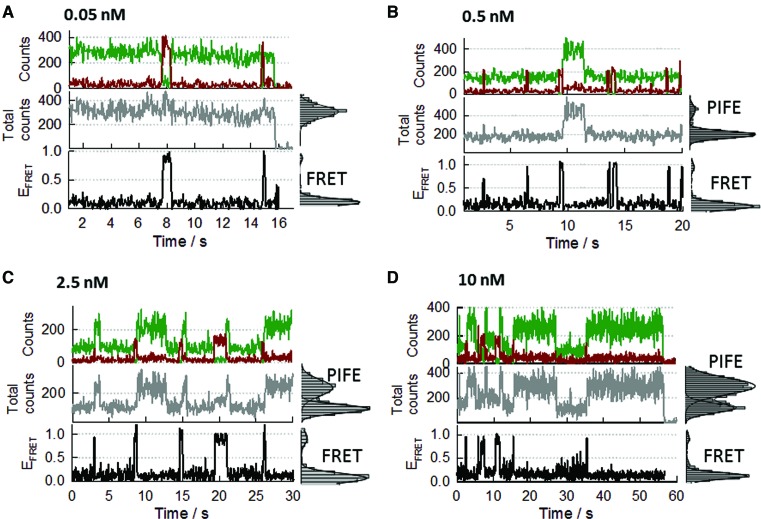
Real-time single-molecule measurements of SsoSSB binding to a 12-mer ssDNA as a function of SsoSSB concentration using an inter-molecular FRET assay. DNA was labelled at the 3′ end with Cy3 and at the 5′ end with a biotin moiety. SsoSSB was site-specifically labelled with Alexa647 as acceptor. Representative single-molecule intensity trajectories of Cy3 and Alexa647 are shown (upper panels) together with the PIFE (middle panel) and the FRET efficiency trajectories (bottom panel) at the indicated concentrations of SsoSSB (**A**: 0.05nM; **B**: 0.5 nM; **C**: 2.5 nM and **D**: 10 nM). Single-molecule population histograms for each FRET and PIFE trajectories are also shown in the right panels. Solid lines represent the corresponding fit of each population to a Gaussian distribution.

Using the above classification to interpret the binding dynamics, we next carried out a single-molecule titration of the surface-immobilized 12-mer strand as a function of SsoSSB concentration (Figure 7). At 0.05 nM concentration of SsoSSB protein (Figure 7A), the trajectories are dominated by the S1 state (donor only signal) with rare fluctuations to the S2a/b state displaying a high FRET value (E_app_∼0.9–1.0). Because the acceptor dye is located on the SsoSSB protein, these bursts of FRET signal are indicative of A647-SsoSSB binding to the immobilized BidC_12_Cy3 strand. The duration of these FRET bursts was very short, lasting in average for less than 0.5 s, suggesting a very transient interaction between SsoSSB monomers and the ssDNA. When the concentration of SsoSSB protein was increased by 10-fold (0.5 nM), the frequency of fluctuations between S1 and S2a-b states increased significantly (Figure 7B and Supplementary Figure S11), thus confirming our assignation of being caused by SsoSSB binding to form BidC_12_Cy3/(A647-SsoSSB) complexes of 1:1 stoichiometry. At this concentration, we detected occasional fluctuations between S2a-b to S3 states, the latter representing the association of two SsoSSB monomers on the BidC_12_Cy3 strand (Figure 7B, middle panel). The frequency of these fluctuations increased considerably at 2.5 nM concentration of SsoSSB (Figure 7C and Supplementary Figure S12) and the average lifetime of the S3 state was ∼10-fold longer than the S2a-b state (∼3–5 s). A further 4-fold increase in SsoSSB concentration resulted in a higher frequency of S2a/b→S3 transitions (Figure 7D and Supplementary Figure S13), which supports our assignation of the S3 state representing Bi-dC_12_Cy3/(A647-SsoSSB)_2_ complexes.

Importantly, the majority of the BidC_12_Cy3/(A647-SsoSSB)_2_ complexes originated following the sequence S1→S2a-b (monomer)→S3 (dimer) and therefore by consecutive monomer by monomer association to the 12-mer strand. Our finding of monomeric SsoSSB as the functional unit dynamically interacting with the ssDNA agrees with a recently reported equilibrium study using cross-linking experiments (60) and with the crystal structure of the OB-fold from SsoSSB (24). SsoSSB behaving mostly as a monomer in solution is further supported by the lack of EPR modulation depth observed at a concentration of SsoSSB of 100 μM (Supplementary Figure S3) and agrees with recently reported size-exclusion chromatography, nuclear magnetic resonance (NMR) and multi-angle laser light scattering data (60) where SsoSSB remained monomeric even at the millimolar range of SsoSSB concentrations required for NMR (60).

### Quantifying SsoSSB binding dynamics to ssDNA

**Figure 8.**
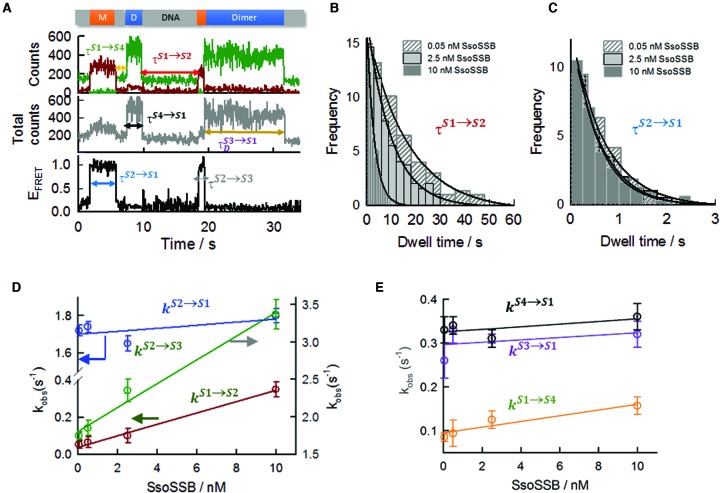
Single-molecule rate analysis of the association and dissociation steps involved in SsoSSB coating of a ssDNA strand. (**A**) Schematic of the dwell-time (τ) assignation of each individual single-molecule state for SsoSSB binding to a 12-mer ssDNA strand. Single-molecule dwell-time histograms for the association (**B**) and dissociation (**C**) of the first bound monomer obtained using a surface-immobilized 12-mer dC single-strand DNA labelled with Cy3 and Biotin (BidC_12_Cy3) at the indicated concentrations of Alexa647 labelled SsoSSB. The association rate $(k^{S1 \to S2} )$ was determined from the dwell-times of the S1 state (no protein bound) and the dissociation rate $(k^{S2 \to S1} )$ was measured as the time a single SsoSSB monomer was bound to the ssDNA and therefore in a high-FRET state (see main text for details). Solid lines represent the results from fitting the dwell-time histograms obtained at each SsoSSB concentration to a mono-exponential decay function. (**D**) Dependence of the pseudo-first order rates for firsrt (dark red) and second monomer association (green) as a function of SsoSSB concentration. The off rate of the first monomer is also shown (blue). The off rate for the first monomer was independent of SsoSSB concentration, while the pseudo-first order rate for the first and second monomer association showed a linear dependence with SsoSSB concentration. For the association rates (dark red and green), the solid lines represent linear fits to yield the second-order association rate constant. (**E**) Association (orange) and dissociation (black) rates obtained at the indicated concentrations of SsoSSB for the low-populated S1→S4 and S4→S1 transitions (S4 pathway, see main text and Figure 6 for details). The solid line represents a linear fit to extract the second-order rate constant. Kinetic rates obtained for the dissociation of ssDNA:(SsoSSB)_2_ complexes formed following the S3 pathway (purple) (see main text and Figure 6 for details) are also shown with their associated errors.

The combination of different photophysical processes (FRET, PIFE and quenching) within the same single-molecule trajectory allowed us to unambiguously assign each dynamic event and determine the binding kinetics as a function of SsoSSB concentration. In Figure 8A, a representative single-molecule trajectory is shown defining all the kinetic rates required to fully determine the binding dynamics to a 12-mer ssDNA. To quantify the association and dissociation kinetics, we determined the dwell times of states S1 to S4 and built cumulative single-molecule dwell-time histograms from >300 trajectories to extract the kinetic rate for each transition. The average dwell time of the unbound state (τ_S1_), which corresponds to the inverse of the pseudo-first order association rate for the first bound monomer, $k^{S1 \to S2}$, was extracted at the different SsoSSB concentrations from the single-molecule histograms of the S1 state (no SsoSSB bound) (Figure 8B). As one would expect for a binary reaction, $k^{S1 \to S2}$ was concentration dependent and showed ∼7-fold increase from 0.05 ± 0.02 s^−1^ (mean ± s.e.m) at 0.05 nM to 0.35 ± 0.04 s^−1^ at 10 nM (Figure 8B). A linear fit of $k^{S1 \to S2}$ against the concentration of SsoSSB yielded a bimolecular association rate constant between the 12-mer strand and the first bound monomer of 0.032 nM^−1^ s^−1^ (Figure 8D). Also as expected, the dwell time of the high FRET S2a-b state (τ_S2_) (Figure 8C), which corresponds to the inverse of the dissociation rate of a single bound monomer, $k^{S2 \to S1}$, was independent on the protein concentration with values of 1.72 ± 0.05 s^−1^ at 0.5 nM and 1.80 ± 0.01 s^−1^ at 10 nM concentration of SsoSSB (Figure 8c). Using these values, the calculated dissociation constant for the first monomer bound (*K*_D_^M1^) was 54 ± 2 nM at 10 mM concentration of KCl. This dissociation constant is close to the 37 nM value obtained by ensemble-FRET methods assuming a two-site binding model (Figure 3B).

The pseudo-first order association rate for the second monomer, $k^{S2 \to S3}$, was extracted from the monoexponential fitting of the single-molecule dwell time histograms of the S2a-b state (Supplementary Figure S14). $k^{S1 \to S2}$ showed only a moderate 2-fold increase from 1.7 ± 0.1 s^−1^ at 0.05 nM to a value of 3.4 ± 0.2 s^−1^ at 10 nM concentration of SsoSSB, yielding a bimolecular association rate of 0.16 ± 0.03 nM^−1^ s^−1^ (Figure 8D). This value is ∼5-fold higher than the association rate observed for the first bound monomer (0.032 nM^−1^ s^−1^), suggesting that binding of the second monomer is favoured at DNA sites adjacent to an already bound monomer. The photobleaching rate of Cy5 and derivatives is known to be strongly enhanced at high incident laser power (58). Thus, to rule out the possibility that the loss of acceptor emission observed for S2→S1 and S2→S3 transitions was significantly contributed by acceptor photobleaching events, we carried out a comparison of the binding dynamics at 2.5 nM concentration of SsoSSB and two excitation conditions differing by more than an order of magnitude in incident laser power (40 W/cm^2^ and 400 W/cm^2^). The rates obtained for S2→S1 and S2→S3 transitions were very similar between both excitation conditions (Supplementary Figure S15). This confirms our assignation of both transitions as being a result of SsoSSB binding dynamics and not photobleaching events.

In contrast to the behaviour observed for BidC_12_Cy3/(A647-SsoSSB) complexes, single-molecule transitions representing the dissociation of one of the two SsoSSB bound monomers from BidC_12_Cy3/(A647-SsoSSB)_2_ complexes were very unusual (<5%) at all the concentrations of SsoSSB investigated. These rare transitions from doubly- to singly-bound ssDNA states were characterized either by the recovery of the FRET signal or by a stepwise decrease in Cy3 emission (Supplementary Figure S16). We interpreted the low percentage of S3→S2a-b transitions detected as evidence that both monomers dissociate simultaneously, or sequentially, but with the dissociation of the first monomer being quickly followed by the second monomer at a rate faster than our time resolution (33 ms). Our data do not allow us to discriminate between both scenarios, but we can still determine a lower limit for BidC_12_Cy3/(A647-SsoSSB)_2_ disassembly leading to uncoated ssDNA $(k^{S3 \to S1} )$ if we assume a single-step dissociation. For this, we fitted the single-molecule dwell-time histogram of the S3 state at each SsoSSB concentration to a mono-exponential decay function (Supplementary Figure S17). $k^{S3 \to S1}$ was independent on SsoSSB concentration and showed an average value of 0.31 s^−1^ (Figure 8D). This rate is ∼6-fold slower than the rate obtained for the dissociation of the BidC_12_Cy3/(A647-SsoSSB) complex (∼1.75 s^−1^), suggesting that the arrival of a second monomer at an adjacent site confers a significant degree of stability. Using the values obtained for the association of the second monomer $(k^{S2 \to S3} )$ and the single-step disassembly of the BidC_12_Cy3/(A647-SsoSSB)_2_ complex, we extracted a dissociation constant for the second monomer (*K*_D_^M2^) of ∼2 nM, indicative of a significant degree of positive cooperativity for this process. This dissociation constant is again very close to the value obtained by ensemble-FRET for an identical dC_12_ strand assuming a two-site binding model (∼2.8 nM).

Although the formation of the S4 state from S1 is a low-populated binding pathway, accounting for only ∼9–17% of the total number of binding events, we determine the kinetics of this process for comparative purposes. The pseudo-first order association rate, $k^{S1 \to S4}$, at each concentration of SsoSSB was obtained from the fitting of the single-molecule dwell-time histograms of S1 states leading to the formation of S4 states (Figure 8E and Supplementary Figure S18a). $k_D^{S1 \to S4}$ exhibited a very small change from 0.085 ± 0.008 s^−1^ at 0.05 nM to a value of 0.16 ± 0.03 s^−1^ at 10 nM concentration of SsoSSB. From the linear fitting of $k^{S1 \to S4}$ against the concentration of SsoSSB, we obtained a bimolecular rate constant of 0.006 ± 0.002 nM^−1^ s^−1^ (Figure 8E). This value is 5-fold slower than rate obtained for S1→S2 transitions (0.032 nM^−1^ s^−1^) and ∼25-fold slower than the rate obtained for the formation of the S3 state from S2 (0.16 nM^−1^ s^−1^). The rate of S4 to S1 disassembly, $k^{S4 \to S1}$, was independent of protein concentration and exhibited a value of 0.33 s^−1^ (Figure 8E and Supplementary Figure S18b). This magnitude is remarkably similar to that obtained for S3 states formed by sequential incorporation of SsoSSB monomers (0.31 s^−1^). From these values we obtained a dissociation constant for the S4 state (*K*_D_) of ∼57 nM.

**Figure 9.**
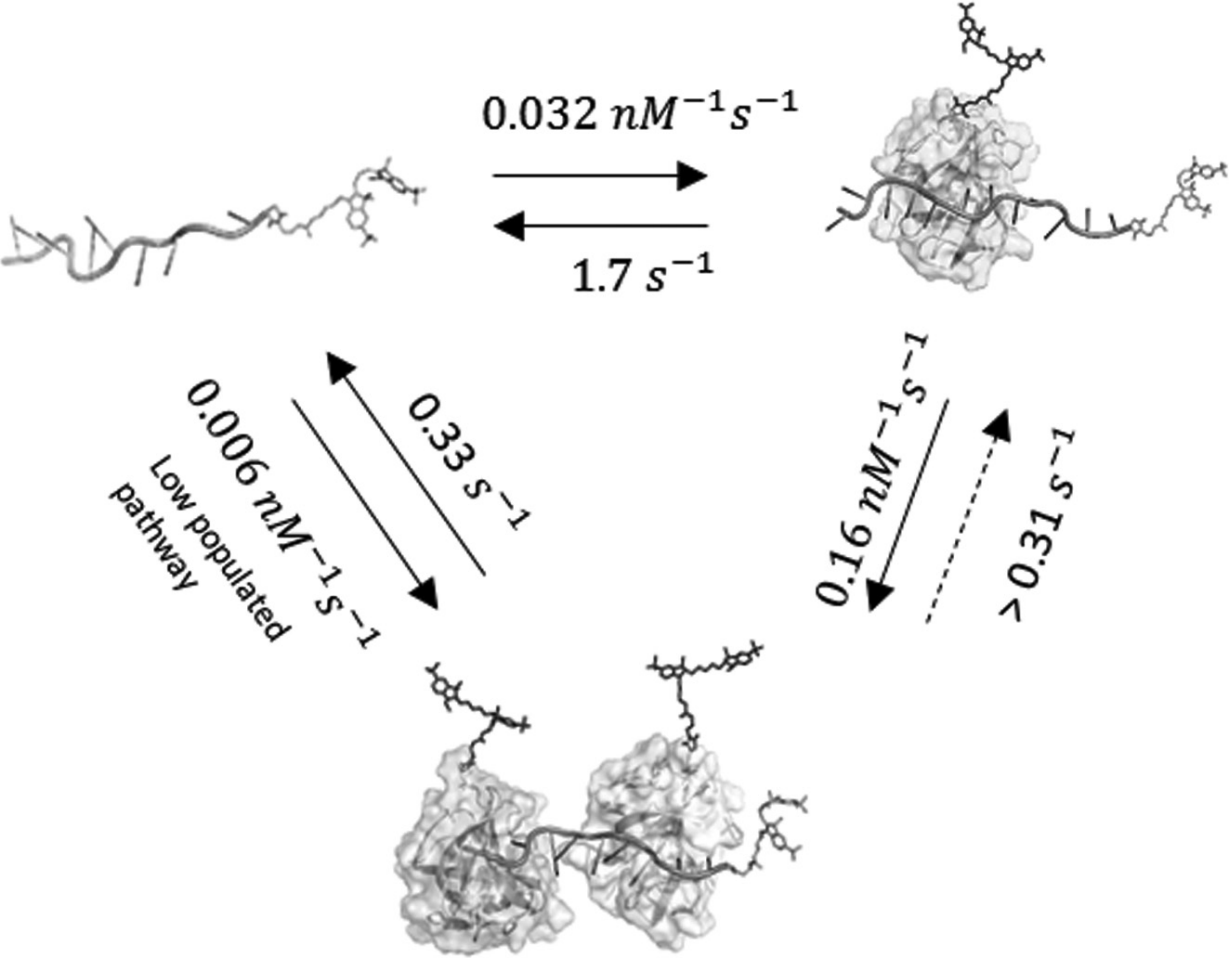
Proposed kinetic model for the interaction of SsoSSB with single-stranded DNA. The cartoon depicts the sequential monomer-by-monomer binding to the SsDNA and the corresponding rates for each step extracted from single-molecule measurements. A low-populated kinetic pathway involving the incorporation of the first monomer to the ssDNA followed by the second monomer at a rate faster than our time resolution (33 ms) is also shown (see main text for details).

As summarized in the scheme in Figure 9, our kinetic analysis confirmed that *S. solfataricus* is predominantly a monomer in solution and indicate that the interaction between SsoSSB and ssDNA is a highly dynamic process that takes place by sequential monomer-to-monomer association to the nucleic acid sequence.

## DISCUSSION

In this study, we have used a combination of photophysical processes to characterize the interaction between *S. solfataricus* single-strand binding protein (SsoSSB) and ssDNA molecules at the ensemble and single-molecule level. Our motivation for this work was dual. Firstly, most of our knowledge of the interaction between single-strand binding proteins and ssDNA is limited to proteins carrying multiple OB-fold domains and very little is known at the single OB-fold level (34–36, 38, 46, 47). Here, the single OB-fold organization of *S. solfataricus* SSB provided an unique platform to fill this gap (60). Secondly, we aimed to expand the range of single-molecule assays to monitor the interaction between SSBs and ssDNA. To date, the majority of single-molecule studies on SSBs relied on monitoring conformational changes in a FRET-labelled ssDNA to indirectly report the binding of SSB proteins (34–36). Furthermore, to facilitate the incorporation of the FRET pair and the biotin moiety required for surface-immobilization, these studies have been restricted to overhang DNA structures. However, it is known that the conformation of the single-stranded DNA is altered by the presence of the dsDNA–ssDNA junction, particularly for short ssDNA fragments, where the duplex DNA excludes the single-stranded DNA chain from its vicinity (61). Due to its intermolecular character, our single-molecule approach reports directly the association of SSB proteins and facilitates the use of only single-stranded DNA fragments. The destabilizing activity at ss-dsDNA junctions reported for certain SSBs such as EcoSSB (4, 5) and *Deinococcus radiodurans* SSB (10) also imposes additional limitations in the use of overhang structures when the primary focus is the protection and stabilization mechanism associated to ssDNA coating by SSBs. Our approach bypasses this limitation and provides a complementary strategy to those assays based on intramolecular FRET. In contrast to most studies on protein–DNA interactions that have employed smFRET and smPIFE in separate single-molecule experiments, we demonstrate, for the first time, the use of both approaches combined with acceptor quenching within the same experiment in a single trajectory. We found this multi-process approach to be a more powerful strategy that allows to unambiguously assign a particular single molecule signal (FRET, PIFE and quenching) to an specific interaction of SsoSSB monomers with the ssDNA. In the future, this multi-process approach could be easily adapted to investigate the filament nucleation, polarity and coating mechanism of other nucleic–acid interacting proteins.

Our ensemble (Figures 1–3) and single-molecule data (Figures 4–8) indicate that two SsoSSB monomers can be efficiently accommodated in a 12-mer ssDNA with nanomolar affinity. Despite its thermophilic character, the dissociation constants differ by <2-fold between room temperature (9 ± 1 nM) (Figure 1A) and 65°C (15 ± 1 nM) (Supplementary Figure S1), suggesting a very moderate temperature dependence. The tight association and binding site size agrees with the observed stoichiometry of 4–5 nt of ssDNA per SsoSSB monomer determined using gel electrophoresis and tryptophan quenching for ssDNA molecules of different length (32), and more recently, by chemical cross-linking and NMR (60). Interestingly, in contrast to early reports on the oligomeric state of SsoSSB in solution and bound to DNA (62), no evidence for the formation of oligomeric structures was found from these studies (60). The lack of PELDOR modulation in the absence of DNA, even at SsoSSB concentrations near 100 μM, additionally confirms that SsoSSB exists mostly as a monomer in solution and that the SsoSSB monomers only associate next to each other when they bind to the same DNA substrate (Supplementary Figure S3). Our single-molecule measurements using Alexa647 labelled SsoSSB and a 12-mer surface-immobilized Cy3-labelled single-stranded DNA provided additional insights into the dynamics of the association process (Figures 5 and 7). With the exception of a 17% of events leading to the complete coating of the 12 mer ssDNA in a single step (S1→S4 transitions), the most commonly observed sequence of events to coat the 12-mer strand followed the order: no-FRET→high-FRET→PIFE and acceptor quenching, corresponding both transitions to the sequential association of the first and second monomer, respectively. This suggests that monomer-by-monomer association is the predominant pathway for the dynamic assembly of SsoSSB proteins onto the ssDNA.

The absence of a multimeric functional form of SsoSSB is also supported by our intramolecular ensemble-FRET assay using a ssDNA length (39 nt) that can accommodate multiple SsoSSBs (Figure 3C and D). Using a similar assay and an overhang DNA structure containing a (dT)_70_ single-stranded DNA region, three wrapping configurations of (dT)_70_ around the multimeric *EcoSSB* have been described: (SSB)_65_, (SSB)_35_ and a low populated (SSB)_35b_ state (63). Each of these configurations was characterized by a distinct high-FRET state corresponding to different degrees of shortening of the end-to-end distance. In contrast, for (dC)_39_ and SsoSSB, we observed a continuous decrease in the end-to-end distance even at SsoSSB concentrations close to 1 μM (Figure 3d). This observation can be justified assuming a model where the accumulation of SsoSSB monomers on the ssDNA next to each other leads to an overall stretching of polymer and thus a decrease in the FRET efficiency. When compared to other SSB proteins extensively studied at single-molecule level such as the above *EcoSSB* (34, 63), but also *DrSSB* (28) or human RPA (53), our data confirm SsoSSB as the only known member of the OB-fold family that exists and functions in solution as a monomer.

Interestingly, PIFE (Figure 1C), acceptor-quenching (Figure 2B) and FRET (Figure 3B) assays performed separately at ensemble level revealed a significant degree of cooperativity between adjacent monomers as indicated by Hill coefficient values between 1.4 and 1.8. Positive cooperativity has been previously observed for the (SSB)_35_ binding mode of EcoSSB where two tetramers bind adjacent to each other on the ssDNA (63), for phage T4 SSB gp32 that has a similar binding size (∼^7^ nt) to SsoSSB (64, 65) and for *Klesiella pneumoniae* SSB (66) and *Salmonella enterica* Serovar Typhimurium LT2 (67). The cooperative character of the interaction between adjacent SsoSSB monomers with the ssDNA was reinforced by analysing the binding dynamics at single-molecule level. Compared to the association (0.032 nM^−1^ s^−1^) and dissociation rates (∼1.75 s^−1^) obtained for the first monomer, the values corresponding to the second monomer were 5-fold faster (0.16 nM^−1^ s^−1^) and 6-fold slower (0.31 s^−1^), respectively. This indicates that cooperativity effects influence both the association rate constant and the lifetime of the complex. From these values we calculated a dissociation constant of ∼2 nM, ∼25-fold lower than the value of 54 nM obtained for the first monomer, confirming that SsoSSB is more likely to bind adjacent to an already bound SsoSSB monomer than to open DNA. The presence of a certain degree of cooperativity was already predicted from isothermal calorimetry measurements (24). From a structural perspective, positive cooperativity can arise from the formation of direct interface contacts between the nearest neighbours, such as the L_45_ loop contacts observed by X-ray crystallography for the (SSB)_35_ binding mode of *EcoSSB* (23), the arginine-mediated interaction motif in *Thermus* SSB (68) or the LAST motif in the T4 gp32 SSB protein (69). Cooperativity can also result from distortion of the adjacent DNA conformation induced by protein binding as suggested for PriB (67) and FOXK1a (70), and indeed several lines of evidence from the X-ray crystal structure of SsoSSB suggested that this might be the mechanism operating for SsoSSB (24). According to this model, cooperativity is likely to result from a reduced entropic penalty for binding to an adjacent occupied site. This protein-induced entropy reduction is caused by a decrease in the number of available conformations of the remaining ssDNA region of the complex compared to ssDNA alone.

A comparison of the association and dissociation rates obtained here for SsoSSB with those reported at similar experimental conditions for SSB homotetramers from *E. coli* (68) and *Mycobacterium tuberculosis* (69) and the heterotrimeric RPA (70) provided new insights regarding the functional dynamics of monomeric versus multimeric SSBs. The association rate obtained for an SsoSSB monomer binding to open ssDNA was 0.032 nM^−1^ s^−1^, which is ∼30-fold lower than the near diffusion-limited association rates of 1.1 nM^−1^ s^−1^ reported for *EcoSSB* binding to (dT)_70_ (71) or 70-fold lower than the values of 2.1 nM^−1^ s^−1^ and 1.6 nM^−1^ s^−1^ reported for the association of human RPA to (dT)_30_ and (dT)_12_ (72), respectively. We speculate that a lower association rate for SsoSSB may result from a higher relative steric hindrance from the C-terminal tail or may be caused by the monomeric structure of the protein itself and the subsequent absence of ‘avidity effects’ present in multimeric proteins. The agreement of the dissociation constant extracted from single-molecule data with that obtained by ensemble methods and with values previously reported (32) rules out the presence of surface-immobilization artifacts influencing the association dynamics. An association rate of 7.9 × 10^3^ M^−1^ s^−1^ has recently been reported for an archaeal nucleic acid binding protein from *Nanoarchaeum equitans* (NeqSSB) (73). NeqSSB shares a 14% identity and a 32% sequence similarity with SsoSSB and potentially functions also as a monomer. However, in contrast to SsoSSB, NeqSSB showed very poor discrimination between single- and double stranded DNA (73).

In addition to slight differences in the association dynamics, the most significant difference between SsoSSB and *EcoSSB* lies on the dissociation rate. *EcoSSB* has been shown to dissociate very slowly, with a rate of 0.0059 s^−1^ at similar salt concentrations as those employed here. This value is ∼300-fold lower than the dissociation rate obtained for the SSoSSB–DNA complex (1.75 s^−1^) and ∼50-fold slower than the rate obtained for the disassembly of the ternary complex (SSoSSB)_2_–DNA (0.3 s^−1^). Interestingly, a fast protein release from the ssDNA is closer to the behaviour observed for RPA, where a rate of 8.6 s^−1^ was obtained using a (dT)_12_ ssDNA substrate (72). Such a short substrate can only support the interaction with the second and third OB-folds of RPA, termed DBD-A and DBD-B (74). This first-stage interaction of RPA with single-stranded DNA has been shown to occlude ∼ 8 nt and displayed a dissociation constant of ∼50 nM, which is strikingly similar to the value obtained here for the association of the first SsoSSB monomer to a 12-mer ssDNA (54 nM). In a second stage, DBD-C and DBD-D RPA domains interact with the neighbouring ssDNA to occlude ∼ 30 nt with a dissociation constant (∼0.05–1 nM) close to the 2 nM value obtained for the cooperative association of an adjacent SsoSSB monomer. In this high affinity-binding mode, RPA dissociates from (dT)_30_ with a much lower rate of 0.06 s^−1.^ Although at a lower degree, ∼5-fold increase in the lifetime of the ternary complex was also observed for SsoSSB.

In view of these analogies, it is possible that the structural similarities observed between apo-SsoSSB and the RPA DBD-B–DNA complex (24) may have a parallel in terms of functional dynamics. The two-step binding model described for human RPA is thought to facilitate the initial interaction of RPA with ssDNA but also allow the displacement of RPA by other DNA-processing proteins (74, 75). In the case of *S. solfataricus*, considering the harsh conditions to which extremophile organisms are exposed (76), a SSB protein acting as a monomer to allow a high-density coating of the ssDNA and thus a more efficient protection against damage, may have evolved as a critical step to ensure DNA integrity and cell survival. However, such tight association needs to be balanced by a quick monomer release to ensure access to specific portions of ssDNA during fundamental cellular processes.

## Supplementary Material

SUPPLEMENTARY DATA

## References

[1] Watson J.D., Crick F.H. (1953). Molecular structure of nucleic acids; a structure for deoxyribose nucleic acid. Nature.

[2] Ciccia A., Elledge S. J. (2010). The DNA damage response: making it safe to play with knives. Mol. Cell.

[3] Branze D., Foiani M. (2008). Regulation of DNA repair throughout the cell cycle. Nat. Rev. Mol. Cell Biol..

[4] Shereda R.D., Kozlov A.G., Lohman T.M., Cox M.M., Keck J.L. (2008). SSB as an organizer/mobilizer of genome maintenance complexes. Crit. Rev. Biochem. Mol. Biol..

[5] Wang G., Vasquez K.M. (2014). Impact of alternative DNA structures on DNA damage, DNA repair, and genetic instability. DNA Repair.

[6] Cubeddu L., White M.F. (2005). DNA damage detection by an archaeal single-stranded DNA-binding protein. J. Mol. Biol..

[7] Ashton N.W., Bolderson E., Cubeddu L., O'Byrne K.J., Richard D.J. (2013). Human single-stranded DNA binding proteins are essential for maintaining genomic stability. BMC Mol. Biol..

[8] Bolderson E., Petermann E., Croft L., Suraweera A., Pandita R.K., Pandita T.K., Helleday T., Khanna K.K., Richard D.J. (2014). Human single-stranded DNA binding protein 1 (hSSB1/NABP2) is required for the stability and repair of stalled replication forks. Nucleic Acids Res..

[9] Richard D.J., Bolderson E., Cubeddu L., Wadsworth R.I., Savage K., Sharma G.G., Nicolette M.L., Tsvetanov S., McIlwraith M.J., Pandita R.K. (2008). Single-stranded DNA-binding protein hSSB1 is critical for genomic stability. Nature.

[10] Eggington J.M., Kozlov A.G., Cox M.M., Lohman T.M. (2006). Polar destabilization of DNA duplexes with single-stranded overhangs by the *Deinococcus radiodurans* SSB protein. Biochemistry.

[11] Richard D.J., Bell S.D., White M.F. (2004). Physical and functional interaction of the archaeal single-stranded DNA-binding protein SSB with RNA polymerase. Nucleic Acids Res..

[12] Wei T., Zhang S., Zhu S., Sheng D., Ni J., Shen Y. (2008). Physical and functional interaction between archaeal single-stranded DNA-binding protein and the 5′-3′ nuclease NurA. Biochem. Biophys. Res. Comm..

[13] Rolfsmeier M.L., Haseltine C.A. (2010). The single-stranded DNA binding protein of Sulfolobus solfataricus acts in the presynaptic step of homologous recombination. J. Mol. Biol..

[14] Mushegian A.R., Koonin E.V. (1996). A minimal gene set for cellular life derived by comparison of complete bacterial genomes. Proc. Natl. Acad. Sci. U.S.A..

[15] Shi H., Zhang Y., Zhang G., Guo J., Zhang X., Song H., Lv J., Gao J., Wang Y., Chen L. (2013). Systematic functional comparative analysis of four single-stranded DNA-binding proteins and their affection on viral RNA metabolism. PLoS One.

[16] Kur J., Olszewski M., Dlugolecka A., Filipkowski P. (2005). Single-stranded DNA-binding proteins (SSBs)-sources and applications in molecular biology. Acta Biochim. Pol..

[17] Drabovich A., Krylov S.N. (2004). Single-stranded DNA-binding protein facilitates gel-free analysis of polymerase chain reaction products in capillary electrophoresis. J. Chromatogr. A.

[18] Perales C., Cava F., Meijer W.J., Berenguer J. (2003). Enhancement of DNA, cDNA synthesis and fidelity at high temperatures by a dimeric single-stranded DNA-binding protein. Nucleic Acids Res..

[19] Arcus V. (2002). OB-fold domains: a snapshot of the evolution of sequence, structure and function. Curr. Opin. Struct. Biol..

[20] Bochkarev A., Pfuetzner R.A., Edwards A.M., Frappier L. (1997). Structure of the single-stranded-DNA-binding domain of replication protein A bound to DNA. Nature.

[21] Fan J., Pavletich N.P. (2012). Structure and conformational change of a replication protein A heterotrimer bound to ssDNA. Genes Dev..

[22] Raghunathan S., Kozlov A.G., Lohman T.M., Waksman G. (2000). Structure of the DNA binding domain of E. coli SSB bound to ssDNA. Nat. Struct. Biol..

[23] Raghunathan S., Ricard C.S., Lohman T.M., Waksman G. (1997). Crystal structure of the homo-tetrameric DNA binding domain of Escherichia coli single-stranded DNA-binding protein determined by multiwavelength x-ray diffraction on the selenomethionyl protein at 2.9-Å resolution. Proc. Natl. Acad. Sci. U.S.A..

[24] Kerr I.D., Wadsworth R.I., Cubeddu L., Blankenfeldt W., Naismith J.H., White M.F. (2003). Insights into ssDNA recognition by the OB fold from a structural and thermodynamic study of Sulfolobus SSB protein. EMBO J..

[25] Suck D. (1997). Common fold, common function, common origin. Nat. Struct. Mol. Biol..

[26] Kozlov A.G., Galletto R., Lohman T.M. (2012). SSB-DNA binding monitored by fluorescence intensity and anisotropy. Methods Mol. Biol..

[27] Antony E., Weiland E., Yuan Q., Manhart C.M., Nguyen B., Kozlov A.G., McHenry C.S., Lohman T.M. (2013). Multiple C-terminal tails within a single E. coli SSB homotetramer coordinate DNA replication and repair. J. Mol. Biol..

[28] Kozlov A.G., Eggington J.M., Cox M.M., Lohman T.M. (2010). Binding of the dimeric Deinococcus radiodurans single-stranded DNA binding protein to single-stranded DNA. Biochemistry.

[29] Wold M.S. (1997). Replication protein A: a heterotrimeric, single-stranded DNA-binding protein required for eukaryotic DNA metabolism. Ann. Rev. Biochem..

[30] Sun S., Shamoo Y. (2003). Biochemical characterization of interactions between DNA polymerase and single-stranded DNA-binding protein in bacteriophage RB69. J. Biol. Chem..

[31] Komori K., Ishino Y. (2001). Replication protein A in *Pyrococcus furiosus* is involved in homologous DNA recombination. J. Biol. Chem..

[32] Wadsworth R.I., White M.F. (2001). Identification and properties of the crenarchaeal single-stranded DNA binding protein from *Sulfolobus solfataricus*. Nucleic Acids Res..

[33] Napoli A., Valenti A., Salerno V., Nadal M., Garnier F., Rossi M., Ciaramella M. (2005). Functional interaction of reverse gyrase with single-strand binding protein of the archaeon Sulfolobus. Nucleic Acids Res..

[34] Zhou R., Ha T. (2012). Single-molecule analysis of SSB dynamics on single-stranded DNA. Methods Mol. Biol..

[35] Zhou R., Kozlov A.G., Roy R., Zhang J., Korolev S., Lohman T.M., Ha T. (2011). SSB functions as a sliding platform that migrates on DNA via reptation. Cell.

[36] Zhang J., Zhou R., Inoue J., Mikawa T., Ha T. (2014). Single molecule analysis of Thermus thermophilus SSB protein dynamics on single-stranded DNA. Nucleic Acids Res..

[37] Blouin S., Craggs T.D., Lafontaine D.A., Penedo J.C. (2009). Functional studies of DNA-protein interactions using FRET techniques. Methods Mol. Biol..

[38] Flynn R.L., Zou L. (2010). Oligonucleotide/oligosaccharide-binding fold proteins: a growing family of genome guardians. Crit. Rev. Biochem. Mol. Biol..

[39] Klare J. P., Roberts GCK (2013). Chemistry of spin labelling. Encyclopedia Biophys.

[40] Okumus B., Wilson T. J., Lilley D. M. J., Ha T. (2004). Vesicle encapsulation studies reveal that single molecule ribozyme heterogeneities are intrinsic. Biophys. J..

[41] McCluskey K., Shaw E.S., Lafontaine D.A., Penedo J.C (2013). Single-molecule fluorescence of nucleic acids. Methods Mol. Biol..

[42] Hwang H., Kim H., Myong S. (2011). Protein induced fluorescence enhancement as a single molecule assay with short distance sensitivity. Proc. Natl. Acad. Sci. U.S.A..

[43] Craggs T.D., Hutton R.D., Brenlla A., White M.F., Penedo J.C. (2013). Single-molecule characterization of Fen1 and Fen1/PCNA complexes acting on flap substrates. Nucleic Acids Res..

[44] Kim J.-Y., Kim C., Lee N.K. (2015). Real-time submillisecond FRET dynamics of freely diffusing molecules with liposome tethering. Nat. Commun..

[45] Rippe K. (1997). Analysis of protein-DNA binding at equilibrium. B.I.F. Futura.

[46] Kernchen U., Lipps G. (2006). Thermodynamic analysis of single-stranded binding activity of the archaeal replication protein A (RPA) from *Sulfolobus Solfataricus*. Biochemistry.

[47] Robbins J.B., Murphy M.C., White B.A., Mackie R.I., Ha T., Cann I.K.O. (2004). Functional analysis of multiple single-stranded DNA-binding proteins from *Methanosarcina acetivorans* and their effects on DNA synthesis by DNA polymerase BI. J. Biol. Chem..

[48] Milov A.D., Salikhov K.M., Shirov M.D. (1981). Application of ENDOR in Electron-spin echo for paramagnetic center space distributions in solids. Fiz. Tverd. Tela..

[49] Pannier M., Veit S., Godt A., Jeschke G., Spiess H. W. (2000). Dead-time free measurement of dipole-dipole interactions between electron spins. J. Magn. Reson..

[50] Ackermann K., Giannoulis A., Cordes D.B., Slawin A.M.Z., Bode B.E. (2015). Assessing dimerization degree and cooperativity in a biomimetic small-molecule model by pulsed EPR. Chem. Commun..

[51] Quinn S.D., Dalgarno P.A., Cameron R.T., Hedley G.J., Hacker C., Lucocq J., Baillie G.S., Samuel I.D.W., Penedo J.C. (2014). Real-time probing of β-amyloid self-assembly and inhibition using fluorescence self-quenching between neighbouring dyes. Mol. Biosys..

[52] Zhoe R., Kunzelmann S., Webb M.R., Ha T. (2011). Detecting intramolecular conformational dynamics of single molecules in short distance range with sub-nanometer sensitivity. Nano Lett..

[53] Nguyen B., Sokoloski J., Galleto R., Elson E.L., Wold M.S., Lohman T.M. (2014). Diffusion of human replication protein A along single-stranded DNA. J. Mol. Biol..

[54] Joo C., McKinney S.A., Nakamura M., Rasnik U., Myong S., Ha T. (2006). Real-time observation of RecA filament dynamics with single-molecule resolution. Cell.

[55] Hutton R.D., Craggs T.D., White M.F., Penedo J.C. (2010). PCNA and XPF cooperate to distort DNA substrates. Nucleic Acids Res..

[56] Stein I.H., Capone S., Smit J.H., Baumann F., Cordes T., Tinnefeld P. (2012). Linking single-molecule blinking and redox potentials. Chemphyschem.

[57] Murphy M. C., Rasnik I., Cheng W., Lohman T.M., Ha T. (2004). Probing single-stranded DNA conformational flexibility using fluorescence spectroscopy. Biophys. J..

[58] Ha T, Tinnefeld P (2012). Photophysics of fluorescence probes for single molecule biophysics and super-resolution imaging. Ann. Rev. Phys. Chem..

[59] Clegg R. M. (1992). Fluorescence resonance energy transfer and nucleic acids. Methods Enzymol..

[60] Roland G., Ruvini K., Adrian X.G., Christine T., Elysse M., Ray E.B., Nicholas E.S., Sandro F.A., Qihan D., Derek J.D. (2015). The structural basis of DNA binding by single-stranded DNA-binding protein from Sulfolobus solfataricus. Biochem. J..

[61] Chen H., Meisburger S.P., Pabit S.A., Sutton J.L., Webb W.W., Pollack L. (2012). Ionic strength-dependent persistence lengths of single-stranded RNA and DNA. Proc. Natl. Acad. Sci. U.S.A..

[62] Haseltine C.A., Kowalczykowski S.C. (2002). A distinctive single-stranded DNA-binding protein from the archaeon sulfolobus solfataricus. Mol. Microbiol..

[63] Roy R., Kozlov A.G., Lohman T.M., Ha T. (2007). Dynamic structural rearrangements between binding modes of E. coli SSB protein. J. Mol. Biol..

[64] Jose D., Weitzel S.E., Baase W, von Hippel P. (2015). Mapping the interactions of the single-stranded DNA binding protein of bacteriophage T4 (gp32) with DNA lattices at single-nucleotide resolution: gp32 monomer binding. Nucleic Acids Res..

[65] Jose D., Weitzel S.E., Baase W.A., Michael M.M., von Hippel P. (2015). Mapping the interactions of the single-stranded DNA binding protein of bacteriophage T4 (gp32) with DNA lattices at single nucleotide resolution: polynucleotide binding and cooperativity. Nucleic Acids Res..

[66] Huang Y.H., Huang C.Y. (2012). Characterization of a single-stranded DNA binding protein from *Klebsiella pneumonia*: mutation at either Arg73 or Ser76 causes a less cooperative complex on DNA. Genes Cells.

[67] Huang C.Y., Hsu C.H., Sun Y.J., Wu H.N., Hsiao C.D. (2006). Complexes crystal structure of replication start primosome protein PriB reveals a novel single-stranded DNA binding mode. Nucleic Acids Res..

[68] Witte G., Fedorov R., Curth U. (2008). Biophysical analysis of *Thermus aquaticus* single-stranded DNA binding protein. Biophys. J..

[69] Casas-Finet J.R., Fischer K.R., Karpel R.L. (1992). Structural basis for the nucleic acid binding cooperativity of bacteriophage T4 gene 32 protein: the (Lys/Arg)3(Ser/Thr)2 (LAST) motif. Proc. Natl. Acad. Sci. U.S.A..

[70] Tsai K.L., Huang C.Y., Chang C.H., Sun Y.J., Chuang W.J., Hsiao C.D. (2006). Crystal structure of the human FOXK1a-DNA complex and its implications on the diverse binding specificity of winged helix/forkhead proteins. J. Biol. Chem..

[71] Kozlov G.A., Lohman T.A. (2002). Stopped-flow studies of the kinetics of single-stranded DNA binding and wrapping the *Escherichia coli* SSB tetramer. Biochemistry.

[72] Patrick S.M., Turchi J.J. (2001). Stopped-flow kinetic analysis of replication protein A-binding DNA. J. Biol. Chem..

[73] Olszewski M., Balsewicz J., Nowak M., Maciejewska N., Cyranka-Czaja A., Zalewska-Piatek B., Piatek R, Kur J. (2015). Characterization of a single-stranded DNA-binding-like protein from *Nanoarchaeum equitans*-A nucleic acid binding protein with broad substrate specificity. PLoS One.

[74] Fan J., Pavletich N.P. (2012). Structure and conformational change of a replication protein A heterotrimer bound to ssDNA. Genes Dev..

[75] Fanning E., Klimovich V., Nager A.R. (2006). A dynamic model for replication protein A (RPA) function in DNA processing pathways. Nucleic Acids Res..

[76] Kelman Z., White M.F. (2005). Archaeal DNA replication and repair. Curr. Opin. Microbiol..

