# Supplementary material for: Binding dynamics of a monomeric SSB protein to DNA: a single-molecule multi-process approach
Source: Nucleic Acids Res. 2015 Nov 17;43(22):10907–24. doi: 10.1093/nar/gkv1225 (PMC4678828; doi:10.1093/nar/gkv1225)
Supplement: SUPPLEMENTARY DATA [file supp_43_22_10907__index.html]

Binding dynamics of a monomeric SSB protein to DNA: a single-molecule multi-process approach — SUPPLEMENTARY DATA 

# Binding dynamics of a monomeric SSB protein to DNA: a single-molecule multi-process approach

## SUPPLEMENTARY DATA

- SUPPLEMENTARY DATA
