# Supplementary material for: Binding dynamics of a monomeric SSB protein to DNA: a single-molecule multi-process approach
Source: Nucleic Acids Res. 2015 Nov 17;43(22):10907–24. doi: 10.1093/nar/gkv1225 (PMC4678828; doi:10.1093/nar/gkv1225)
Supplement: SUPPLEMENTARY DATA [file supp_gkv1225_NAR_Morten_et_al_2015_Supplementary_revised_version.pdf]

Binding dynamics of a monomeric SSB protein to DNA: a single-molecule multi-process approach

Michael J. Morten[1,4], Jose R. Peregrina[1], Maria Figueira-Gonzalez[1,5], Katrin Ackermann[1,2], Bela E. Bode[1,2], Malcolm F. White[,1] and J. Carlos Penedo[*,1,3]*

[1]Biomedical Sciences Research Complex, University of St Andrews, St Andrews, Fife, United Kingdom, KY16 9ST; [2]EaStCHEM School of Chemistry and Centre of Magnetic Resonance, University of St Andrews, St Andrews, Fife, United Kingdom, KY16 9ST; [3]SUPA School of Physics and Astronomy, University of St Andrews, St Andrews, Fife, United Kingdom, KY16 9SS

* To whom correspondence should be addressed
jcp10@st-andrews.ac.uk
mfw2@st-andrews.ac.uk

Present Address:
[4]School of Chemistry, The University of Glasgow, Joseph Black Building, University Avenue, Glasgow, G12 8QQ, United Kingdom.
[5] Faculty of Chemistry, University of Santiago de Compostela, E-27002, Spain.

Supplementary Methods

Ensemble fluorescence assays

PIFE experiments were performed by exciting the Cy3 dye at 545 nm and calculating the emission area from 560 to 650 nm. The concentration of 12-mer ssDNA was 10 nM and the KCl concentration was 10 mM. PIFE measurements were performed at room temperature (20 °C) and 65 °C. The calculated Cy3 emission area at each concentration of SsoSSB was normalized with respect to the emission area in the absence of protein. Data were fitted using non-linear squares to a Hill binding model described by Equation S1:

$$\frac{F_{SSB}}{F_o} = \frac{B_{max}X^{\alpha}}{(K_D^{\alpha} + X^{\alpha})} \qquad \text{[Equation S1]}$$

where B_{max} is the maximum specific binding, K_D is the concentration required for half-maximum binding, and α is the Hill coefficient.

Quenching experiments were performed by exciting Alexa647-SsoSSB at 645 nm and recording the fluorescence spectra from 650 to 800 nm. The percentage of Alexa647 quenching at each concentration of 12-mer DNA added was obtained by comparing the emission area at each DNA concentration with respect to that in the absence of DNA. The concentration of SsoSSB was 10 nM and the KCl concentration was 10 mM. All quenching experiments were performed at room temperature. The binding isotherm was fitted to a Hill binding model using Equation S1 (1).

Inter-molecular FRET experiments were performed exciting the Cy3 donor (λ_{exc} 545 nm) as above and the energy transfer efficiency was calculated using the donor quenching method as described by Equation S2:

$$E_{FRET} = 1 - \frac{I_D^A}{I_D} \qquad \text{[Equation S2]}$$

where I_D^A and I_D represent the sum of the emission intensities in the presence and absence of acceptor (Alexa647), respectively. Two titrations were performed in order to separate variations in the emission of the donor due to changes in FRET or due to PIFE. The variation in FRET efficiency was also fitted to a two-site model described by Equation S3:

$$F_{obs} = \frac{B_1^{max}K_1\,[SSB]}{K_1 + [SSB]} - \frac{B_2^{max}K_2[SSB]}{K_2 + [SSB]} \qquad \text{[Equation S3]}$$

where F_{obs} correspond to the FRET signal, B_1^{max} and B_2^{max} are the values associated with one and two SsoSSB monomers bound, respectively. K_1 and K_2 are the stepwise dissociation equilibrium constants for the binding of the first monomer (K_1) leading to efficient energy transfer and the second monomer (K_2) leading to acceptor quenching and loss of the FRET signal.

Intra-molecular FRET measurements were performed under magic angle conditions to avoid anisotropy artifacts and analysed by exciting the donor dye, Cy3 at 545 nm and recording the emission spectrum from 560 to 800 nm. The acceptor dye (Alexa647) emission spectrum was also recorded using an excitation wavelength of 645 nm and emission monitored from 650 to 800 nm. The efficiency

of energy transfer was calculated using the RatioA method because using this method variations in the donor and acceptor quantum yield due to protein binding do not interfere with the estimation of the FRET efficiency (2).

PELDOR measurements

All PELDOR data were recorded as previously described (3). With the exception of the following timings all settings and optimisation procedures were used as described previously (4). The pump pulse was set to 20 ns at X-band and 12 – 14 ns at Q-band, τ_1 to 380 ns, τ_2 to 3 - 4 μs at X-band and 6 μs at Q-band, and the shot repetition time to 2.5 - 3 ms, averaging the data for approximately 12 h at X-band or < 3.5 h at Q-band. Raw PELDOR data were subjected to background correction assuming a monoexponential decay, followed by Tikhonov regularisation in DeerAnalysis2013 (5). Resulting distance distributions were validated with the validation tool within DeerAnalysis2013, using a noise level of 1.50 (noise increased by 50% over the experimental noise) and 5 trials, and varying the background start time from 5% to 95% of the total data acquisition time window in 6 trials, resulting in 30 trials in total. A prune level of 1.15 was applied, i.e. retaining only data sets within 1.15 times of the best root mean square deviation. If less than 50% of the trials remained upon pruning (SSB at X-band and SSB + 9A at Q-band) data were cut by 10% and subsequently by 5% until more than 50% of the trials remained. This procedure eliminated validations dominated by distortions of the raw data at long dipolar evolution times. Shown in the validation figures are the $\pm 2 \times \sigma$ confidence intervals as coloured shaded areas.

Molecular modelling of the SSB:DNA complex

The crystallographic structure 1JMC of RPA70 with single stranded DNA (1JMC) was used for generating the model. A new strand of 9C was generated using the 8C DNA in 1JMC as template, preserving the structure of the 3 first and 3 last nucleotides. Later, two SsoSSB structures (1O7I) were added aligning them with corresponding position of the RPA domains respect to the DNA. CCP4 and PyMol were used in this process. The structure was protonated and immersed in a rectangular water box using sodium atoms as counter-ions. The energy of whole system was minimized, and finally it was equilibrated up to 298 K. NAMD with Amber force-field parameters was used for these later steps.

References

1. Rippe, K. (1997) Analysis of protein-DNA binding at equilibrium. *B. I. F. Futura*, **12**, 20-26.
2. Blouin, S., Craggs, T.D., Lafontaine, D.A and Penedo, J. C (2009) Functional Studies of DNA-Protein Interactions Using FRET Techniques *Methods in Molecular Biology*, **543**, 475-502.
3. Kerry, P.S., Turkington, H.L., Ackermann, K., Jameison, S.A.,Bode, B.E. (2014) Analysis of influenza A virus NS1 dimer interfaces in solution by pulse EPR distance measurements. *J. Phys. Chem. B*, **118***,* 10882-10888.
4. Bode, B.E., Margraf, D., Plackmeyer, J., Durner, G., Prisner, T.F., Schiemann, S. (2007) Counting the monomers in nanometer-sized oligomers by pulsed electron-electron double resonance. *J. Am. Chem. Soc.*, **129**, 6736–6745
5. Jeschke, G., Chechik, V., Ionita, P., Godt, A., Zimmermann, H., Banham, J., Timmel, C.R., Hilger, D., Jung, H. (2006) DeerAnalysis2006-a comprehensive software package for analysing pulsed ELDOR data. *Appl. Magn. Reson*., **30**, 473-498.

Supplementary Figures

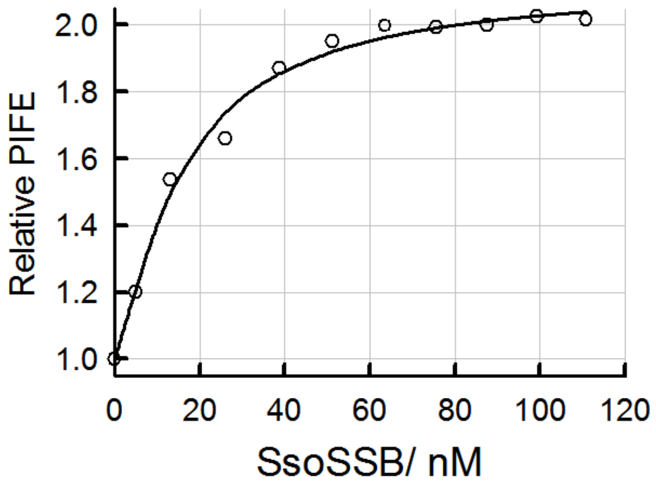

Figure S1. SsoSSB binding to a 12-mer Cy3-labelled single-strand DNA at 65° C monitored by protein induced fluorescence enhancement (PIFE). The solid line represents the fit to a Hill model (Equation S1). We obtained values of 15 ± 1 nM and 1.4 ± 0.2 for the dissociation constant and the Hill coefficient, respectively.

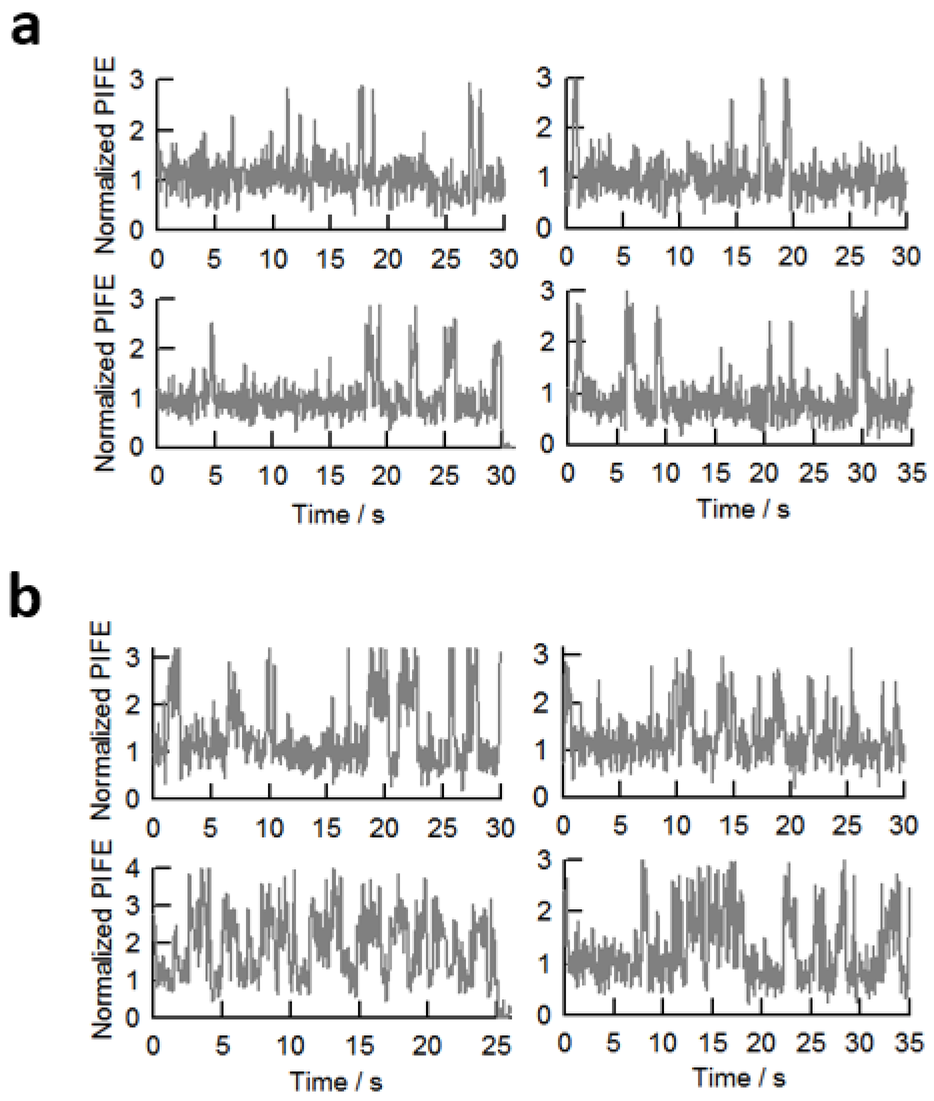

Figure S2. Representative single-molecule PIFE trajectories obtained at 1 nM (a) and 10 nM (b) concentration of SsoSSB obtained with 50 ms integration time. SsoSSB association and dissociation can be observed as PIFE events in the single molecule trace. The Cy3 intensity in the absence of SssoSSB has been normalized to unity and this has been taken as the signal reference for the single-molecule trajectories to quantify the relative increase in Cy3 intensity due to SsoSSB binding

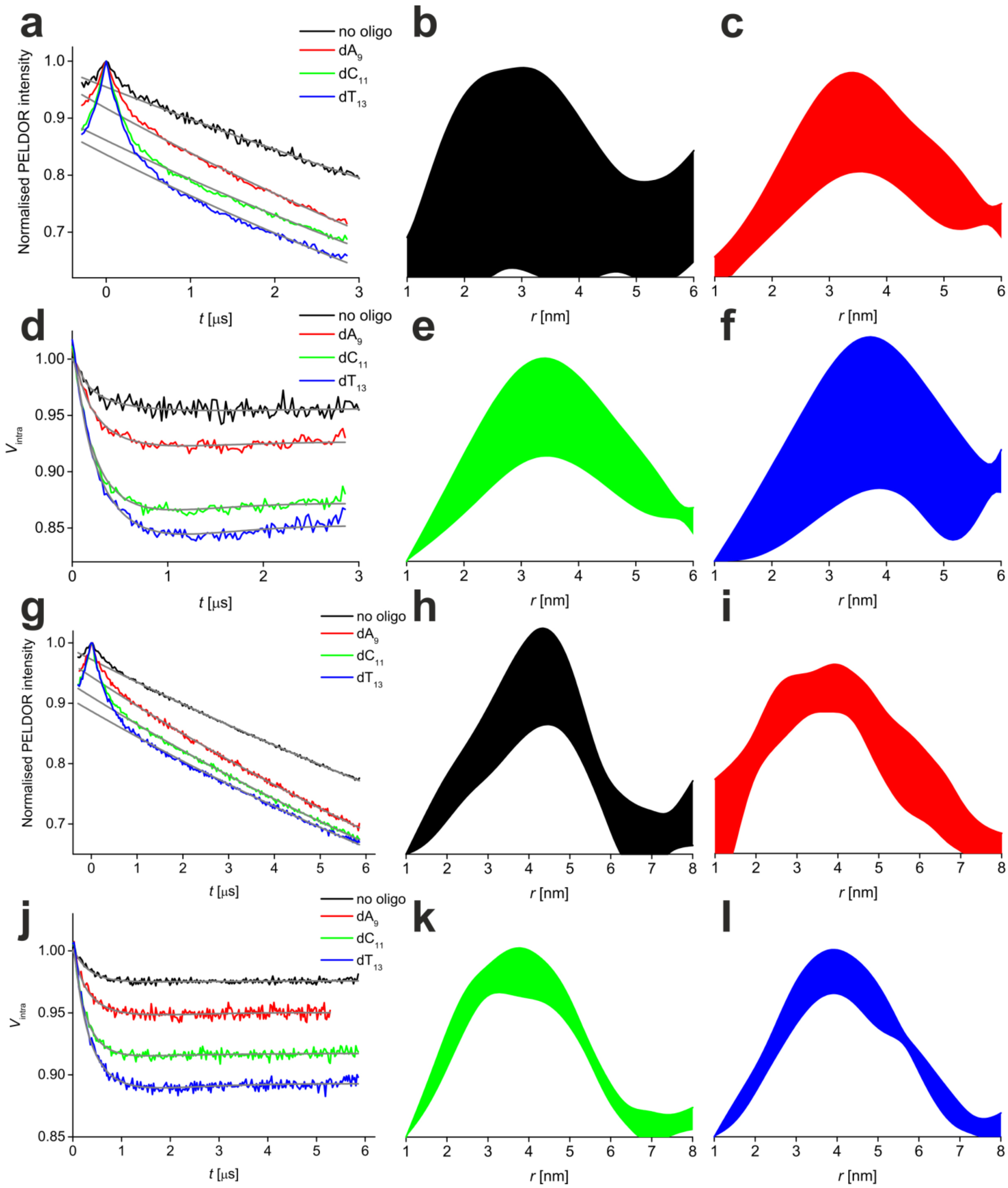

Figure S3. PELDOR analysis of SsoSSB organization in the presence and absence of ssDNA at X- and Q-band given in (a) – (f) and (g) – (l), respectively. (a) and (g) show the raw data before (black) and after A114R1 SsoSSB was introduced to dA_9 (red), dC_{11} (green) or dT_{13} (blue) ssDNA. The background-corrected data in (d) and (j) (same colors as (a) and (g)) demonstrate the observed increase in modulation depth upon addition of ssDNA to A114R1 SsoSSB. Approximating a modulation depth parameter of λ = 0.4 at X-band and 0.25 at Q-band ~90% of the species under observation without ssDNA were monomers. The data from the addition of ssDNA was consistent with increasing formation of dimers that made up ~40% of the A114R1 SsoSSB in solution in presence of dT_{13}. Distance distributions between the spin labels in A114R1 SsoSSB dimers in absence of ssDNA (b) and (h), with added dA_9 (c) and (i), dC_{11} (e) and (k) and dT_{13} (f) and (l) show the ± 2 σ confidence interval as coloured area. These results are consistent with a modelled most probable distance of 4.1 nm.

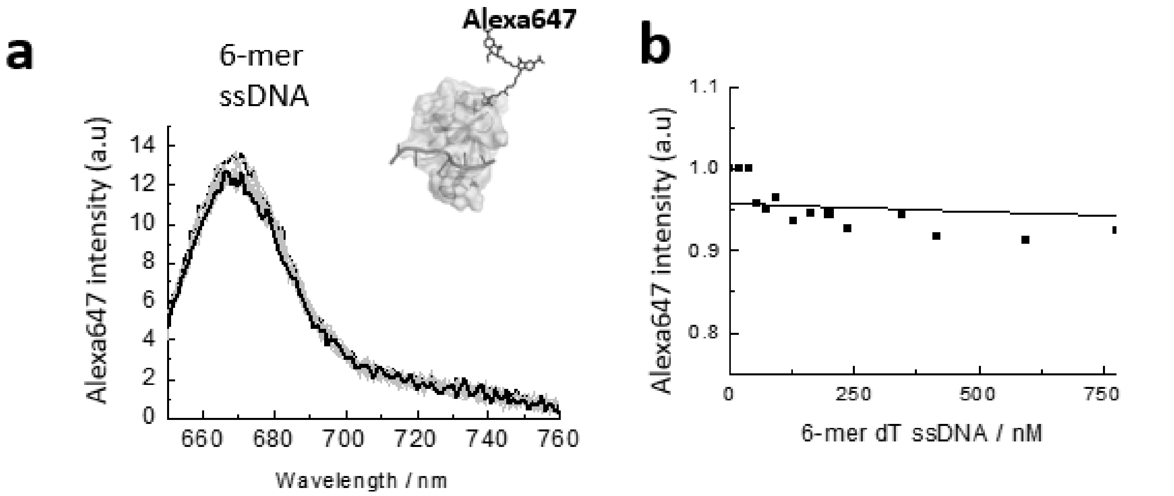

Figure S4. Quenching of Alexa Fluor 647 takes place only upon adjacent binding of two SsoSSB monomers. Fluorescence spectra of SsoSSB labelled with Alexa647 obtained at increasing concentrations of a 6-mer dT single-strand DNA that can only accommodate a single monomer (a) and corresponding emission values recorded at the emission maxima of Alexa647 for each DNA concentration (b). No significant change in the fluorescence intensity was observed suggesting that the formation of a SsoSSB:DNA complex of stoichiometry 1:1 does not influence the photophysis of the dye.

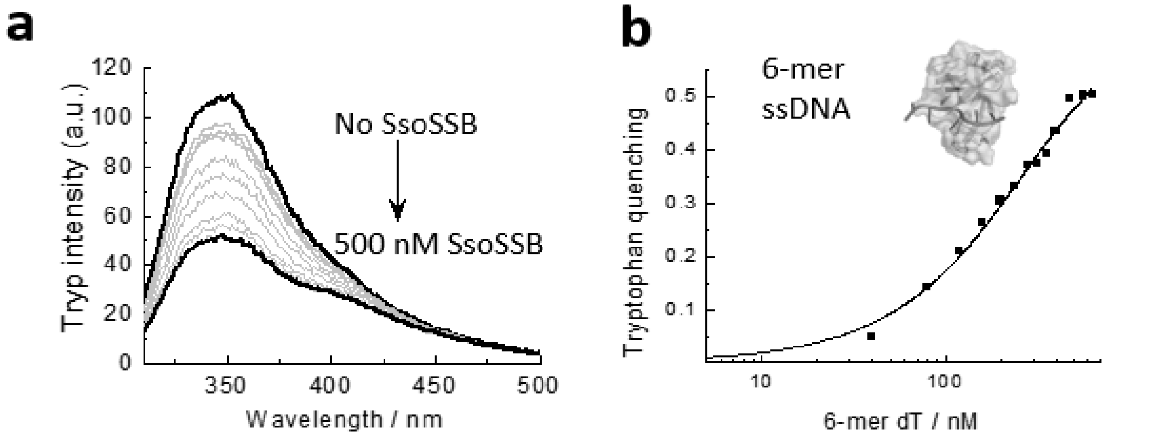

Figure S5. (a) Fluorescence spectra of Tryptophan at increasing concentrations of 6-mer dT ssDNA. (b) Binding isotherm of SsoSSB to a 6-mer dT ssDNA. The solid line represents the fit to a Hill binding equation. This data indicate that the absence of quenching observed a 6-mer dT segment (Figure S4) is not due to the lack of binding.

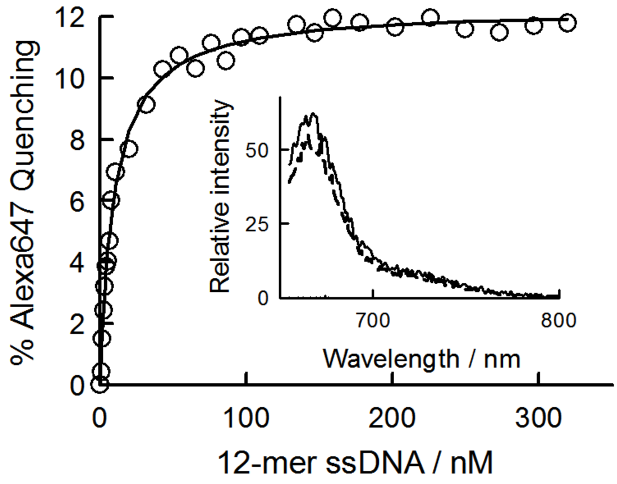

Figure S6. Quenching of Alexa Fluor 647 decreases in mixtures of labelled and unlabelled SsoSSB. SsoSSB binding to a 12-mer single-strand DNA at room temperature monitored by Alexa Fluor 647 quenching. Binding isotherm obtained for the association of a mixture of 5 nM A647SsoSSB and 50 nM unlabelled SsoSSB to ssDNA as a function of ssDNA concentration. The quenching reaches a plateau at ~ 12% quenching compared to ~80% when using only A647-SsoSSB (see main text). Inset: variation in fluorescence spectra in the absence of ssDNA (solid line) and with 300 nM of ssDNA (dashed line). The solid line represents a fit to a Hill model (Eq. S1). Values of 12 ± 2 nM and 1.2 ± 0.3 were obtained for the dissociation constant and the Hill coefficient, respectively.

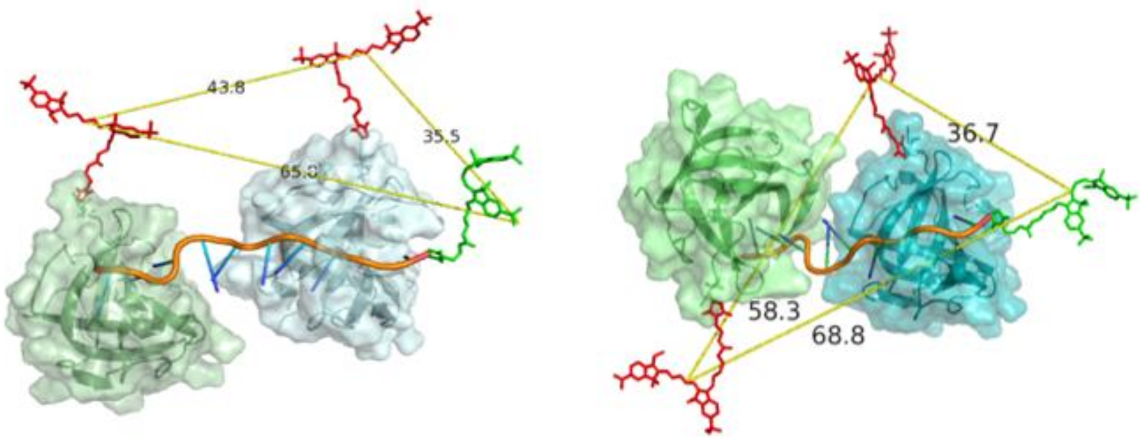

Figure S7. Two possible modelled orientations of the DNA:(SsoSSB)$_2$ complex generated as described in the Supplementary Methods section. The models include the position of the Alexa647 acceptor (red) on the SsoSSB and the position of the Cy3 donor (green) on a 12 mer ssDNA. Estimated values for inter-dye distances in angstroms are also given.

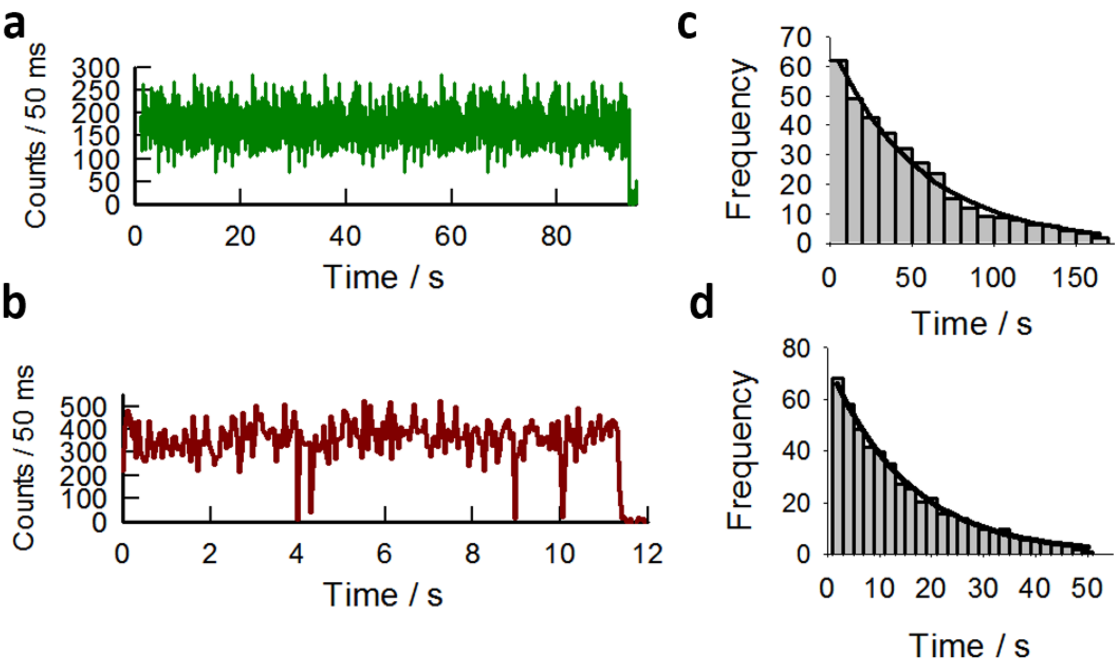

Figure S8. Representative single molecule intensity trajectory of Cy3 incorporated at the 3' end of a 12-mer ssDNA and immobilized in a quartz slide (a) and a representative single molecule intensity trajectory of directly excited A647-SsoSSB (b) encapsulated in a 100 nM diameter eggPC vesicles prepared using the extrusion method (see Materials and Methods section in main text). (c) Single-molecule dwell-time histograms of Cy3 photobleaching at 200 W/cm^2. The solid line represents a fit to a monoexponential decay giving a photobleaching rate of 0.018 ± 0.002 s^{-1}. (d) Single-molecule dwell-time histogram of Alexa Fluor 647 photobleaching at 200 W/cm^2. The solid line represents a fit to a monoexponential decay giving a photobleaching rate of 0.07 ± 0.01 s^{-1}.

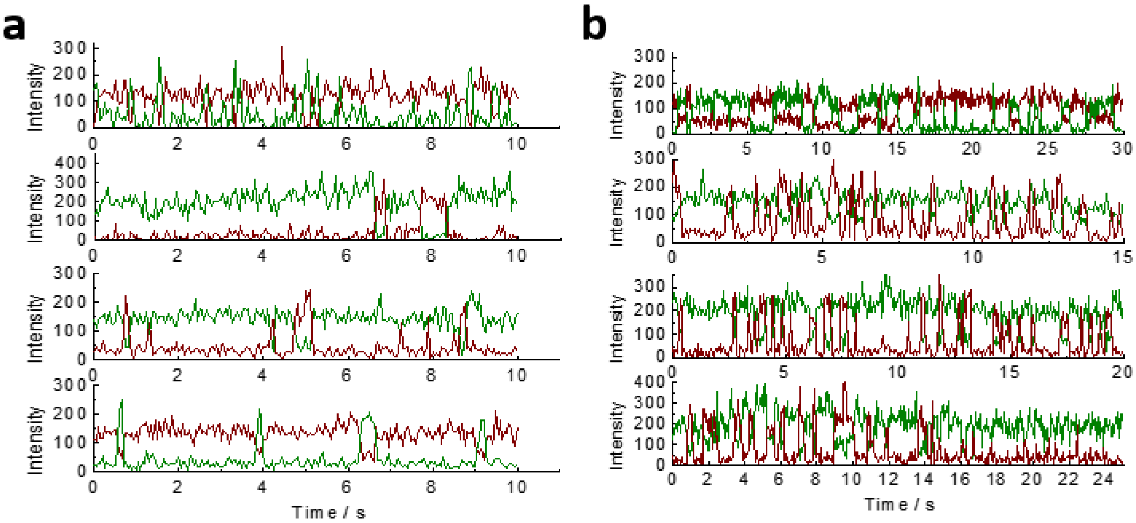

Figure S9. Representative single-molecule donor (green) and acceptor (dark red) intensity trajectories obtained using a Cy3-labelled BidC$_{20}$Cy3 surface-immobilized DNA strand at 3 nM (a) and 30 nM (b) concentration of Alexa647-SsoSSB in a background of 10 mM KCl. Anticorrelated fluctuations in the donor and acceptor signal are indicative of efficient energy transfer due to SsoSSB binding. Integration time was 50 ms.

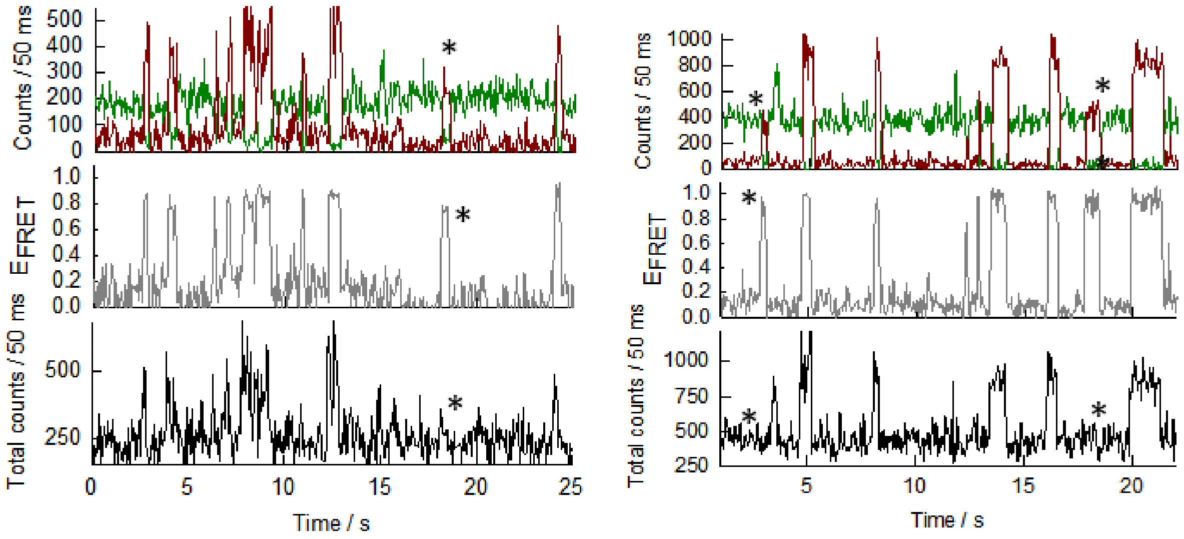

Figure S10. Representative single-molecule donor (green) and acceptor (dark red) intensity trajectories obtained using a Cy3-labelled BidC12Cy3 surface-immobilized DNA strand at 10 nM concentration of A647-SsoSSB in a background of 150 mM KCl. Anticorrelated fluctuations in the donor and acceptor signal are indicative of efficient energy transfer due to SsoSSB binding. Integration time was 50 ms. Two groups of high-FRET states are observed where the total intensity remains constant (S2a state, marked with an asterisk) or increases due to PIFE (S2b state). The latter is the most frequently populated at high ionic strength.

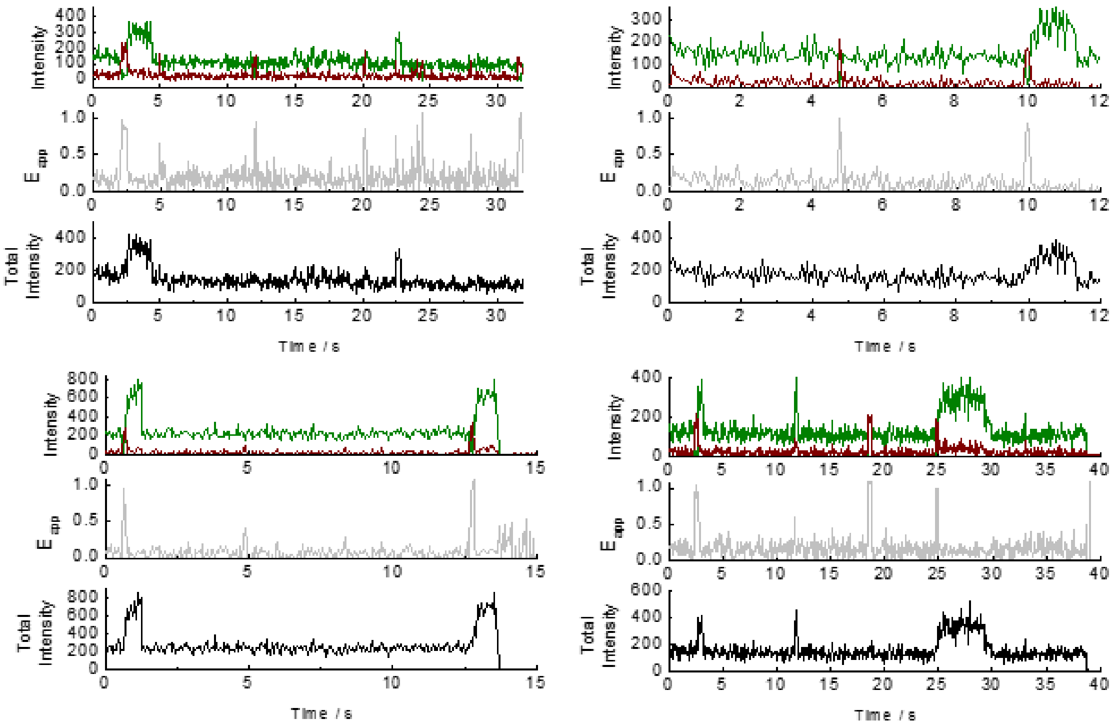

Figure S11. Representative single-molecule measurements including Cy3 (green) and Alexa647 (dark red) (upper panel) emission intensities, FRET efficiency (middle panel) and total intensity (bottom panel) obtained using a Cy3-labelled BidC12Cy3 surface-immobilized DNA strand and 0.5 nM concentration of Alexa647 labelled SsoSSB. Integration time was 50 ms and KCl concentration was 10 mM.

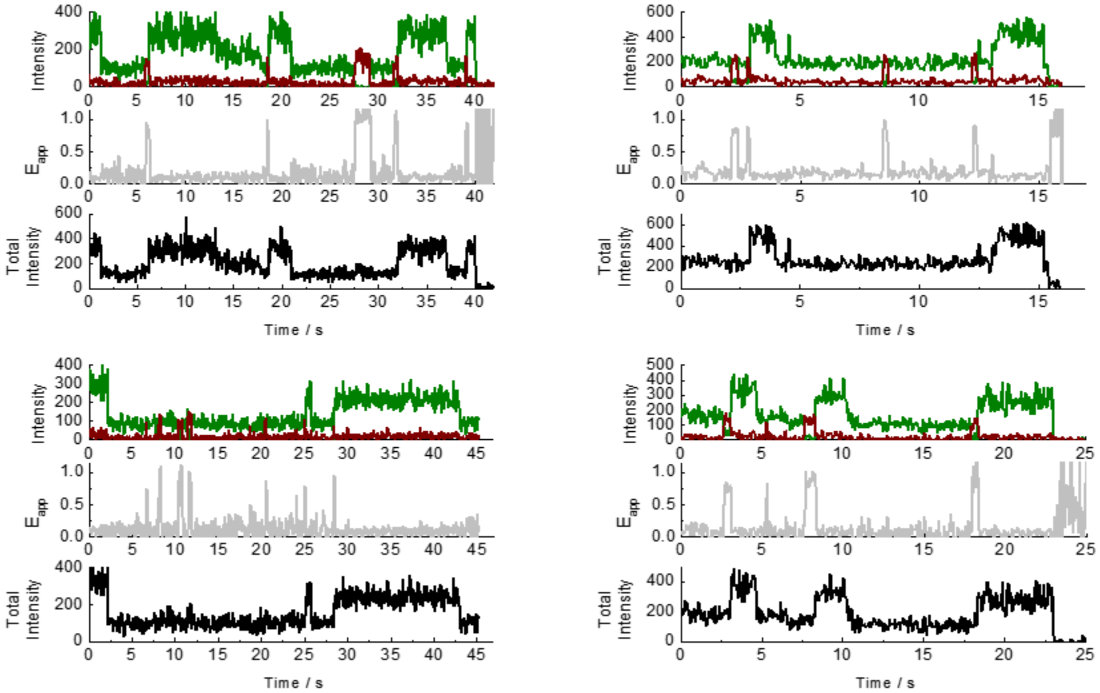

Figure S12. Representative single-molecule measurements including Cy3 (green) and Alexa647 (dark red) (upper panel) emission intensities, FRET efficiency (middle panel) and total intensity (bottom panel) obtained using a Cy3-labelled BidC12Cy3 surface-immobilized DNA strand and 2.5 nM concentration of Alexa647 labelled SsoSSB. Integration time 50 ms and KCl concentration was 10 mM.

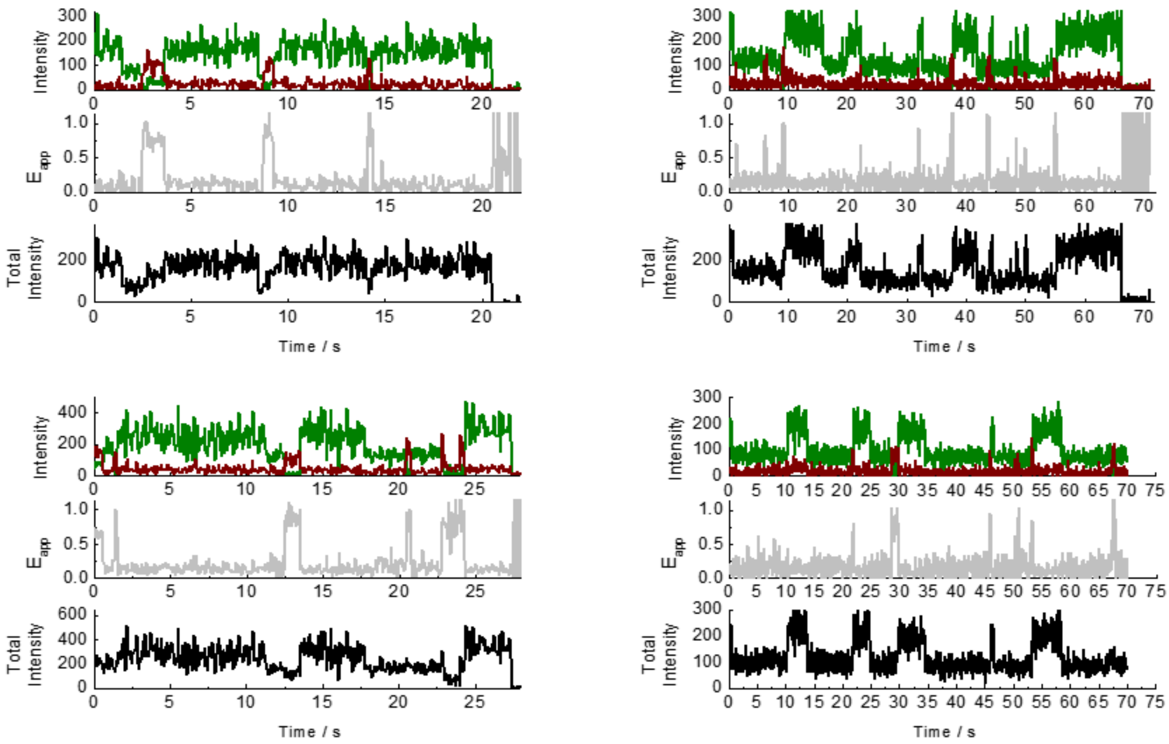

Figure S13. Representative single-molecule measurements including Cy3 (green) and Alexa647 (dark red) (upper panel) emission intensities, FRET efficiency (middle panel) and total intensity (bottom panel) obtained using a Cy3-labelled BidC12Cy3 surface-immobilized DNA strand and 10 nM concentration of Alexa647 labelled SsoSSB. Integration time 50 ms and KCl concentration was 10 mM.

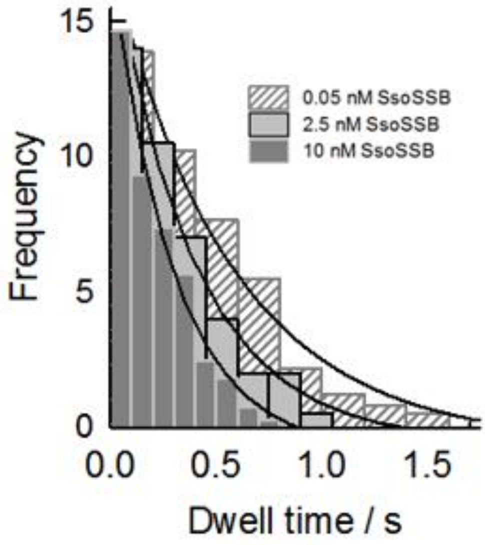

Figure S14. Single-molecule dwell-time histograms for the association (a) of the second monomer to a surface-immobilized 12-mer dC single-strand DNA labelled with Cy3 and Biotin (BidC12Cy3) and the indicated concentrations of Alexa647 labelled SsoSSB. The association rate ($k^{S2\rightarrow S3}$) was determined from the dwell-time of FRET states leading to PIFE events (see main text for details). Solid lines represent the result from fitting the dwell-time histograms to a mono-exponential decay function to extract each the association rate at each concentration.

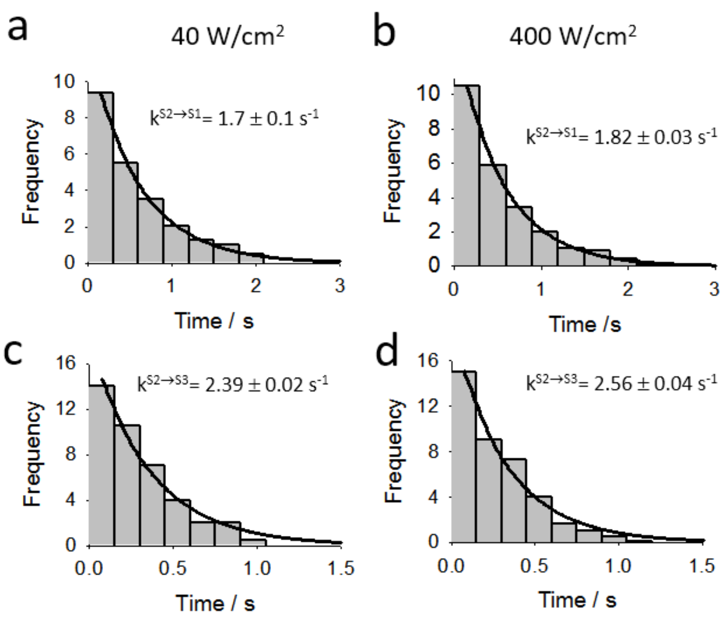

Figure S15. The dwell time of the high-FRET state representing single monomer dissociation events does not depend on the excitation power. Single-molecule dwell-time histograms obtained at 40 W/cm^2 (left panels) and 400 W/cm^2 (right panels) for the S2→S1 transition (a, b) and the S2→S3 transition (c, d). The rates obtained from a monoexponential fitting of each histogram are also shown. The concentration of A647SsoSSB was 2.5 nM.

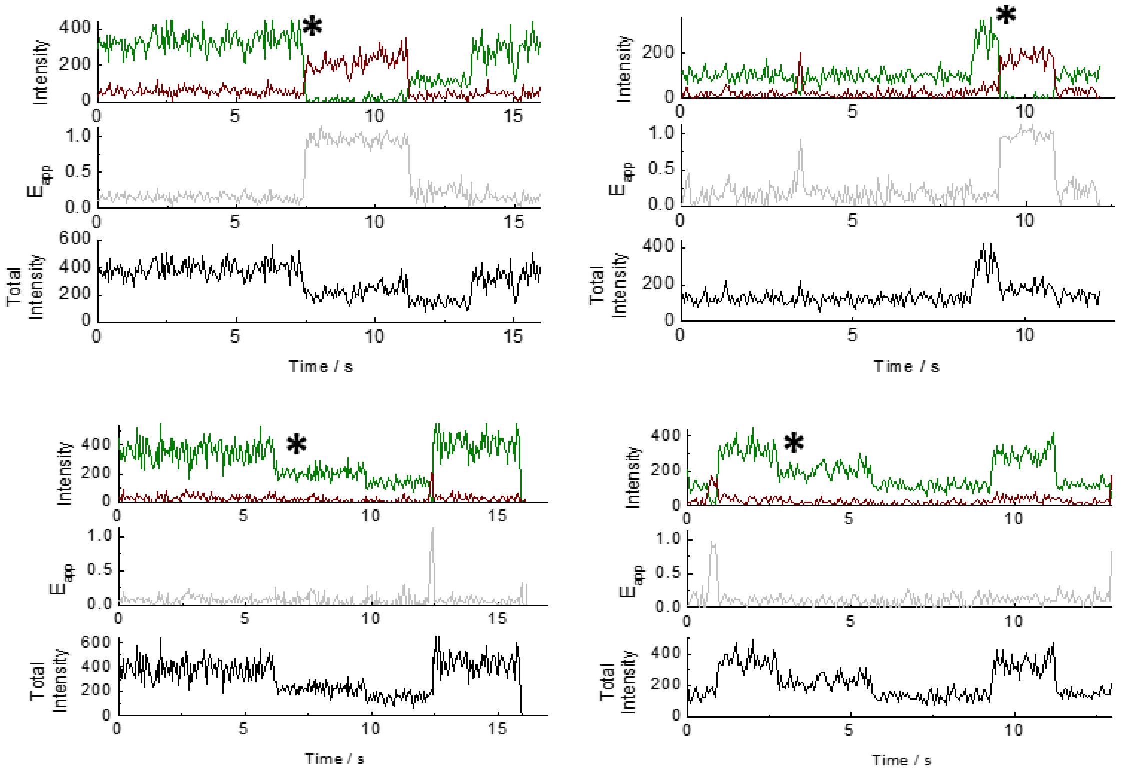

Figure S16. Representative single-molecule donor (green) and acceptor (dark red) intensity trajectories (upper panels), FRET trace (middle panels, grey) and total intensity (bottom panels, black) showing stepwise disassembly events of the $(SsoSSB)_2$:DNA complex marked with an asterisk. Upper row: disassembly events taking place as transitions from PIFE to FRET states corresponding to dissociation of a single monomer and the subsequent disruption of acceptor quenching. Lower row: disassembly events taking place as a stepwise decrease in Cy3 intensity corresponding to loss of PIFE due to single monomer dissociation. Both types of disassembly events are very rare and represent less than 15% of the total number of dissociation events.

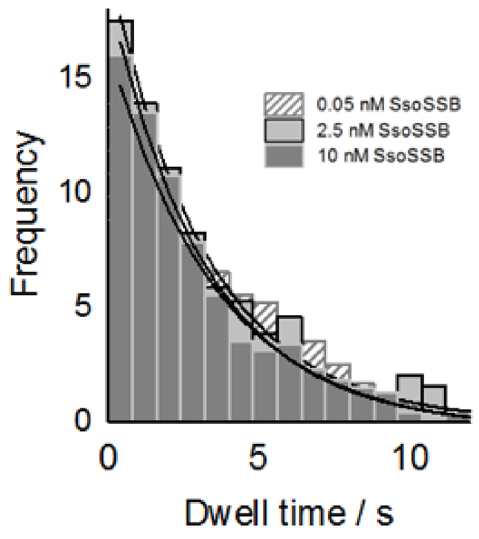

Figure S17. Single-molecule dwell-time histograms obtained for the dissociation of $(SsoSSB)_2$:DNA complexes ($k^{S3\rightarrow S1}$) formed by monomer-to-monomer incorporation leading to free ssDNA at the indicated concentrations of SsoSSB. Solid lines represent the results from monoexponential fitting of the dwell-time histogram to extract the dissociation rate at the indicated concentrations of SsoSSB.

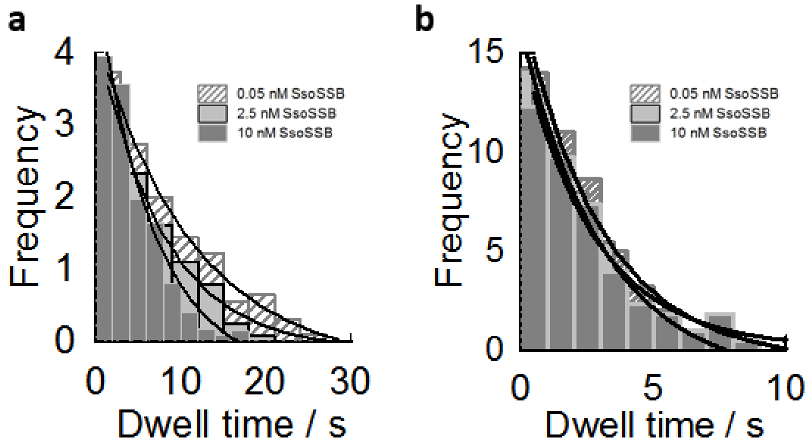

Figure S18. Single-molecule dwell-time histograms obtained for the association (a) of both monomers to a surface-immobilized 12-mer dC single-strand DNA in a single step (S1→S4) at the indicated concentrations of SsoSSB. The association rate ($k^{S1\rightarrow S4}$) was determined from the dwell-time of unbound states (S1) directly leading to PIFE events (see main text for details). Solid lines represent the result from fitting the dwell-time histograms to a mono-exponential decay function to extract each the association rate at each concentration. (b) Single-molecule dwell-time histograms obtained for the dissociation of $(SsoSSB)_2$:DNA complexes ($k^{S4\rightarrow S1}$). Only those complexes formed following a single S1→S4 step at the indicated concentrations of SsoSSB were taken for this analysis. Solid lines represent the results from monoexponential fitting of the dwell-time histogram to extract the dissociation rate at the indicated concentrations of SsoSSB.
